# Immunoengineered MXene nanosystem for mitigation of alloantigen presentation and prevention of transplant vasculopathy

**DOI:** 10.1016/j.nantod.2022.101706

**Published:** 2023-02

**Authors:** Weiang Yan, Alireza Rafieerad, Keshav Narayan Alagarsamy, Leena Regi Saleth, Rakesh C. Arora, Sanjiv Dhingra

**Affiliations:** a Institute of Cardiovascular Sciences, St. Boniface Hospital Albrechtsen Research Centre, Department of Physiology and Pathophysiology, Max Rady College of Medicine, Rady Faculty of Health Sciences, University of Manitoba, Winnipeg, Manitoba R2H 2A6, Canada; b Section of Cardiac Surgery, Department of Surgery, Max Rady College of Medicine, Rady Faculty of Health Sciences, University of Manitoba, Winnipeg, Manitoba R3E 0W2, Canada

**Keywords:** Ti_3_C_2_T_x_ MXene, Nanomedicine, *In vivo* immunomodulation, Allograft vasculopathy, Tissue regeneration

## Abstract

MXenes are an emerging class of nanomaterials with significant potential for applications in nanomedicine. Amongst MXene technologies, titanium carbide (Ti_3_C_2_T_x_) nanomaterials are the most mature and have received significant attention to tackle longstanding clinical challenges due to its tailored physical and material properties. Cardiac allograft vasculopathy is an aggressive form of atherosclerosis and a major cause of mortality among patients with heart transplants. Blood vessel endothelial cells (ECs) stimulate alloreactive T-lymphocytes to result in sustained inflammation. Herein, we report the first application of Ti_3_C_2_T_x_ MXene nanosheets for prevention of allograft vasculopathy. MXene nanosheets interacted with human ECs and downregulated the expression of genes involved in alloantigen presentation, and consequently reduced the activation of allogeneic lymphocytes. RNA-Seq analysis of lymphocytes showed that treatment with MXene downregulated genes responsible for transplant-induced T-cell activation, cell-mediated rejection, and development of allograft vasculopathy. In an *in vivo* rat model of allograft vasculopathy, treatment with MXene reduced lymphocyte infiltration and preserved medial smooth muscle cell integrity within transplanted aortic allografts. These findings highlight the potential of Ti_3_C_2_T_x_ MXene in treatment of allograft vasculopathy and inflammatory diseases.

## Highlights

• A new MXene-based nano-microsystem was designed to prevent transplant rejection.
• MXene was internalized by human endothelium and changed its immunologic behavior.
• MXene treatment improved tolerance of endothelial cells by allogeneic lymphocytes.
• MXene-loaded human endothelial cells reduced interferon signaling in lymphocytes.
• Intravascular MXene reduced *in vivo* rejection of transplanted tissues in a rat model.

## Graphical Abstract

**Figure:**
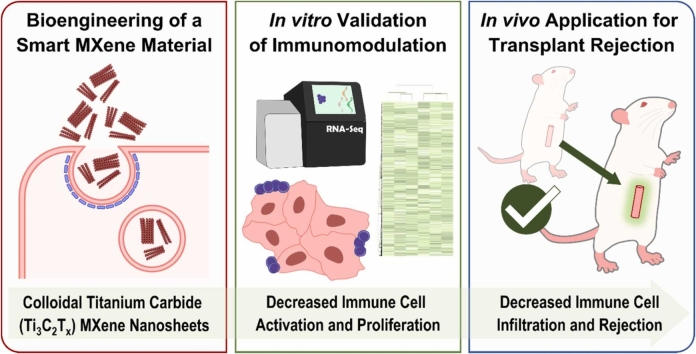

## Introduction

In the last 10–15 years, there has been significant progress in nanotechnology towards designing functional biomaterials for various therapeutic applications [1], [2], [3]. This includes application of organic-inorganic functional nanomaterials with different structures, including single, hybrid, composite, and hetero-structures [4], [5], [6], [7], [8], [9]. More recently, the ongoing development of low-dimensional carbon materials has given rise to novel immunomodulatory platforms for regenerative nanomedicine [10], [11], [12], [13]. These functional biomaterials can form unique, intimate, and complex interactions with human cells and tissues, and offer unprecedented opportunities to tackle longstanding clinical challenges. In particular, recently discovered two-dimensional (2D) transition metal carbide, MXene, has garnered significant attention in multiple biomedical fields. These materials possess high surface areas enriched with biologically active surface groups to promote cellular and tissue interactions with the material itself. Among MXenes, titanium carbide (Ti_3_C_2_T_x_) has received significant attention for applications in nanomedicine due to its tailored physical and material properties. In particular, pioneering applications of Ti_3_C_2_T_x_ MXene nanomaterials have been successfully reported for drug delivery, antimicrobial therapy, cancer therapy, biosensors, radiographic imaging, and bioelectronics. In several studies, Ti_3_C_2_T_x_ MXene has significantly outperformed existing classes of biomaterials [14], [15], [16]. Furthermore, recent reports on the antiviral activity of Ti_3_C_2_T_x_ MXene nanosheets against SARS-CoV2 has invigorated interest in the immunomodulatory behaviour of this material [16]. The ability of MXenes to generate targeted immune responses creates significant hope for future applications of Ti_3_C_2_T_x_ MXene nanosheets in immunoengineering-based nanomedicine.

In patients with end-stage heart failure heart transplantation currently remains the gold standard treatment [17]. However, survivors after heart transplantation face an ongoing risk of complications from organ rejection [18]. In particular, cardiac allograft vasculopathy (CAV), or transplant coronary artery disease, is an aggressive form of accelerated atherosclerosis and a major cause of long-term morbidity and mortality amongst patients with heart transplants. CAV manifests as diffuse concentric intimal thickening of both the micro- and macrovasculature in the transplanted heart. The disease can be clinically silent and the first presentation is often myocardial infarction, arrhythmia, or sudden cardiac death [19]. While its pathogenesis is multifactorial, inflammation plays a key role in the development and evolution of CAV [20], [21]. Currently established treatments for CAV are largely ineffective, and new immunoengineering-based approaches may hold the key to reducing this disease burden [22].

To this regard, the current study reports the first evaluation and application of Ti_3_C_2_T_x_ MXene nanosheets for treatment of allograft vasculopathy and future translational applications. We have comprehensivly characterized the microstructure and surface chemistry of two-dimensional Ti_3_C_2_T_x_ MXene nanosheets. Our studies suggest that titanium carbide nanosheets are highly biocompatible and bioactive. Importantly, Ti_3_C_2_T_x_ MXene nanosheets can effectively interact with human endothelial cells (ECs) to regulate alloantigen presentation and cellular adhesion. These ECs subsequently regulate immune cells and reduce lymphocyte activation, proliferation, and interferon signaling. Furthermore, our data demonstrate that treatment with Ti_3_C_2_T_x_ MXene nanosheets can mitigate immune cell infiltration and allograft injury when applied in an *in vivo* model of cardiac allograft vasculopathy. These findings support that Ti_3_C_2_T_x_ MXene nanosheets possess innate immunomodulatory properties that have potential application for immune-sensitive therapies in regenerative medicine and organ transplant rejection.

## Results and discussion

### Physicochemical properties of Ti_3_C_2_T_x_ MXene nanosheets

**Fig. 1:**
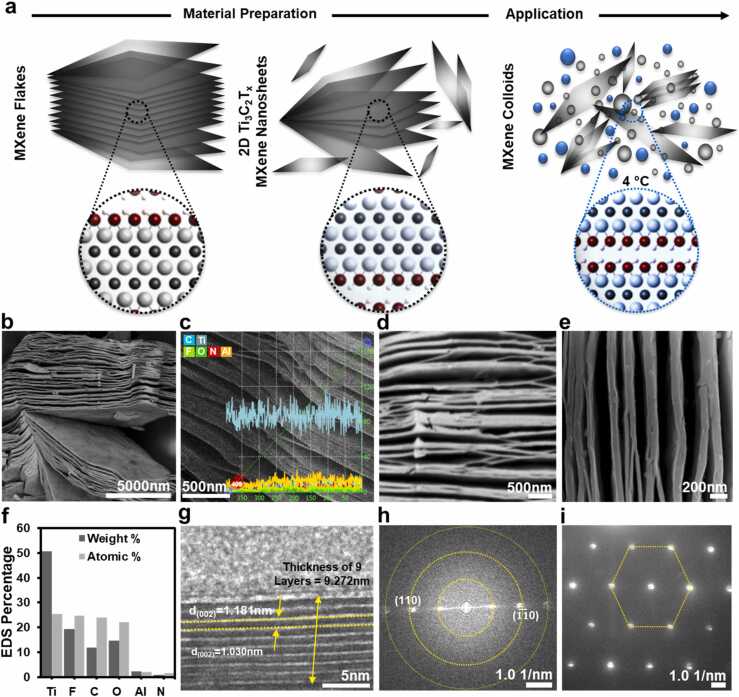
Preparation and microstructural characterization of Ti_3_C_2_T_x_ MXene nanosheets. **a** Schematic model of the preparation processes of two-dimensional Ti_3_C_2_T_x_ nanosheets. **b** SEM images of the MXene nanosheets revealed proper separation of the layers with interlayer spacing of around 150 nm. **c** EDS line scan of MXene nanosheets showing elemental composition of this material. Titanium, carbon, oxygen, and fluorine were the dominant elements. **d**,**e** Backscattered electron and SEM images of as-prepared Ti_3_C_2_T_x_ MXene nanosheets. **f** EDS area scan of the Ti_3_C_2_T_x_ MXene nanosheets was performed to quantify the atomic and weight percentage of different elements in the structure of MXene flakes. **g** High-resolution TEM images of MXene nanosheets demonstrated that the thickness of 9 layers of Ti_3_C_2_T_x_ MXene nanosheets is 9.272 nm. **h**,**i** The FFT and SAED analysis of Ti_3_C_2_T_x_ MXene nanosheets confirmed a hexagonal crystalline structure of MXene nanosheets.

A schematic model depicting the preparation and atomic structure of titanium carbide MXene nanosheets is shown in Fig. 1a. Sonication treatment of Ti_3_C_2_T_x_ flakes dispersed in saline solution resulted in preparation of a colloidal suspension containing of mono-, oligo-, and multi-layered MXene nanosheets. Next, we performed thorough microstructural characterization of Ti_3_C_2_T_x_ MXene nanosheets. Biological performance of a nanomaterial is fundamentally determined by its composition and structure. Biomaterials leverage favourable material-cell and material-tissue interactions to elicit desirable physiologic responses. In particular, differences in the elemental distribution, delamination, and surface functionalization of MXenes have been shown to produce varying biologic effects [16]. An in-depth understanding of these physicochemical properties is therefore essential to both infer mechanisms and to ensure reproducibility of biomaterial activity. Therefore, we performed a detailed physicochemical characterization of Ti_3_C_2_T_x_ MXene nanosheets as described below.

The scanning electron microscopy (SEM) and energy-dispersive X-ray spectroscopy (EDS) analysis of Ti_3_C_2_T_x_ material exhibited well-defined morphological and structural characteristics of MXene nanosheets. The SEM micrographs of the material confirmed the two-dimensional structure of Ti_3_C_2_T_x_ MXene nanosheets with effective separation of layers (Fig. 1b). Furthermore, EDS line scan of MXene flakes demonstrated that titanium, carbon, oxygen, and fluorine were the main elements in the structure of Ti_3_C_2_T_x_ MXene (Fig. 1c). The backscattered electron micrograph of Ti_3_C_2_T_x_ nanosheets in Fig. 1d further reflected the elemental composition of a homogeneous and planar structure. Additionally, representative cross SEM image of Ti_3_C_2_T_x_ MXene exhibited formation of nanosheets with an average wall-to-wall space of ⁓150 nm, which is in line with previous literature (Fig. 1e) [23], [24]. These findings were corroborated by the EDS area scan and mapping analysis, which displayed similar elemental compositions (Fig. 1f and Supplementary Fig. S1). The EDS spectrum of Ti_3_C_2_T_x_ MXene showed atomic percentages of 25.47, 24.64, 23.98, 22.14, and 2.13 for titanium, fluorine, carbon, oxygen, and aluminum, respectively. Additionally, the detection of nitrogen in the EDS spectrum (1.64 at%) can be attributed to the presence of amine-containing functional groups on the surface of MXene.

Next, we characterized the microstructure of Ti_3_C_2_T_x_ MXene using transmission electron microscopy (TEM) and found that MXene nanosheets showed a well-defined planar geometry with interlayer space ranging from 1.030 nm to 1.181 nm. Furthermore, the crystallographic pattern of Ti_3_C_2_T_x_ nanosheets, as detected by fast Fourier transform (FFT) and selected area electron diffraction (SAED) analysis, confirmed the typical hexagonal crystalline structure of MXene (Fig. 1g). Of note, the FFT analysis in Fig. 1h suggested that the dominant crystal planes of Ti_3_C_2_T_x_ were attributed to the (110) planes. Additionally, the diffraction spots in the SAED pattern of Ti_3_C_2_T_x_ also confirmed the hexagonal crystalline planes of MXene nanosheets. Together, these data suggest that these Ti_3_C_2_T_x_ nanosheets possess the microstructural characteristics of MXene material.

**Fig. 2:**
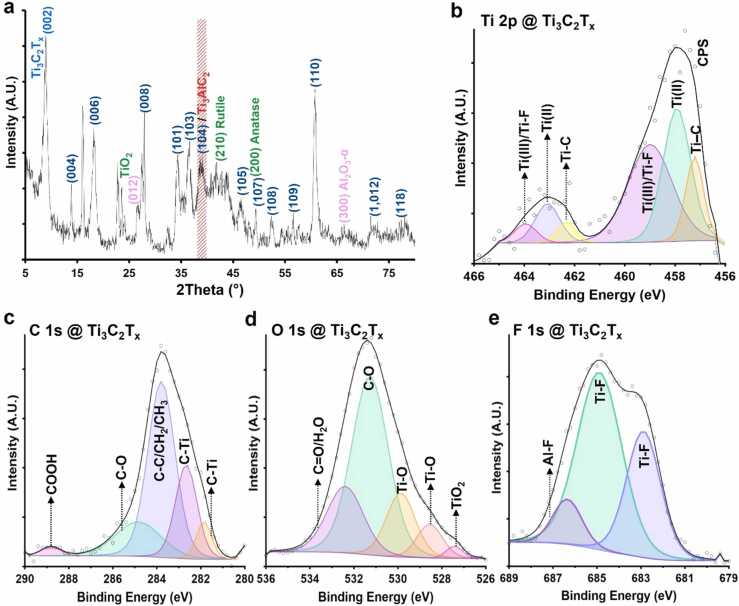
Phase characterization and surface chemistry of Ti_3_C_2_T_x_ MXene nanosheets. **a** The XRD analysis of Ti_3_C_2_T_x_ MXene showing phase pattern of nanosheets. The XRD data demonstrated the presence of characteristic (002) peak at 2-theta of ⁓8º and significant downshift in the dominant titanium aluminum carbide peak at ⁓39°, suggesting negligible presence of aluminum in the composition of Ti_3_C_2_T_x_ MXene nanosheets. **b**-**e** The XPS narrow scan spectra of Ti 2p, C 1s, O 1s, and F 1s corresponding to Ti_3_C_2_T_x_ MXene powder. Our XPS analysis showed typical characteristics of a two-dimensional material and agrees with the XRD data. These findings confirmed that the Ti_3_C_2_T_x_ nanosheets were successfully synthesized and were enriched with the surface functional groups including —OH, COOH, C—O/C=O, and fluorine-based terminals. These surface functional groups are responsible for improving the biocompatibility and bioactivity of Ti_3_C_2_T_x_ MXene nanosheets.

The x-ray diffraction (XRD) analysis of Ti_3_C_2_T_x_ MXene revealed crystalline structure of nanosheets with negligible presence of aluminum layers (Fig. 2a). In particular, a peak at approximately 8° 2-theta reflects the characteristic (002) plane of MXene nanosheets. Additionally, we also detected minor peaks in the XRD pattern of nanosheets which suggested the formation of titanium oxide (TiO_2_) nanocrystals on the edge of MXene nanosheets. This might be due to surface oxidation of the MXene nanosheets when powders were exposed to air during sample preparation for XRD measurement [25], [26]. Furthermore, the presence of small aluminium oxide peaks in the structure of MXene nanosheets suggested possible reaction of remaining traces of aluminum to form stable alpha-phase alumina. Notably, the absence of reactive aluminum atoms in the Ti_3_C_2_T_x_ nanosheets is reported to improve the biocompatibility of MXene materials [27], [28].

Next, we analyzed the surface chemistry of Ti_3_C_2_T_x_ MXene nanosheets using high-resolution x-ray photoelectron spectroscopy (XPS). Data from the XPS analysis indicated the typical surface characteristics of Ti_3_C_2_T_x_ MXene nanosheets. The narrow scan spectrum of Ti 2p revealed dominant peaks of Ti−C, Ti−O, and Ti−F at binding energies ranging from 456 eV to 466 eV (Fig. 2b). The XPS fitting in the Ti 2p spectrum of MXene nanosheets also contained two dominant peaks of titanium oxide, Ti (II) and Ti (III), at the binding energies of 458.03 eV and 459.1 eV, respectively. The C 1s spectrum of Ti_3_C_2_T_x_ MXene was fitted with five peaks (Fig. 2c). The first two peaks at binding energies of 281.85 eV and 282.66 eV corresponded to the titanium carbide MXene bonds (Ti−C or C−Ti) with atomic concentrations of 6.23% and 22.2%, respectively. The other three peaks are mainly assigned to graphitic C–C owing to carbon bonds and oxygen-containing functional groups formed on the MXene surface. Further, our C 1s XPS narrow scan analysis demonstrated the presence of C−C/C−H, C−O, and COOH peaks with atomic concentrations of 52.35%, 17.27%, and 1.94% at binding energies of 283.79 eV, 284.86 eV, and 288.82 eV, respectively. Notably, the carbon-contamination bonds or graphitic carbon in the structure of Ti_3_C_2_T_x_ MXene nanosheets may have formed due to partial etching of titanium layers during the synthesis process [29]. Additionally, minor presence of these carbon-contamination peaks on MXene surface may also be due to adventitious deposition during storage or XPS sample preparation [30], [31]. The O 1s narrow scan XPS spectra and curve fitting of Ti_3_C_2_T_x_ nanosheets contained four dominant peaks corresponding to titanium carbide MXene and oxygen-containing titanium compounds (Fig. 2d). In particular, Ti−O peaks with atomic concentrations of 7.27% and 15.64% were identified at 528.55 eV and 529.87 eV. Additionally, the XPS composition of O 1s included two peaks of C−O and C=O/H_2_O at binding energies of 531.27 eV and 532.40 eV, referring to both Ti_3_C_2_T_x_ and the water molecules between MXene flakes. Also, a minor peak (1.68% atomic concentration) at 527.43 eV was detected which can be attributed to the oxidation state of titanium components in the structure of MXene nanosheets [32], [33], [34].

The XPS narrow scan of Ti_3_C_2_T_x_ MXene nanosheets at the F 1s region identified dominant peaks at binding energies of 682.88 eV and 684.88 eV, assigned to Ti−F bonds in the composition of MXene nanosheets (Fig. 2e). This XPS analysis demonstrated that the highest atomic concentration in the F 1s spectrum belongs to Ti−F peaks (90.23%). Furthermore, narrow scan XPS fitting of this region showed a minor peak of Al−F at 686.35 eV with less than 10% of total atomic concentration at F 1s spectrum. Further details of XPS peak positions and quantifications of as-synthesized MXene nanosheets are listed in Supplementary Tables S1 to S4. Together, these data confirmed the presence of hydroxyl, carboxyl, as well as fluorine-based functional groups on the surface of Ti_3_C_2_T_x_ MXene nanosheets [35].

### Optimization of the biocompatibility of Ti_3_C_2_T_x_ MXene nanosheets

After implantation of a biomaterial in the body, the vascular endothelium plays a critical role in maintaining favourable biomaterial-tissue interactions. In particular, for colloidal biomaterials, the monolayer of endothelial cells can directly facilitate biomaterial uptake, retention, and activity through regulation of inflammation, thrombogenesis, and vascular permeability. Furthermore, endothelial injury and activation during or after allograft transplantation can accelerate the pathogenesis of cardiac allograft vasculopathy by upregulating expression of cellular adhesion molecules, recruitment of immune cells, release of cytokines, and ultimately smooth muscle cell proliferation [36], [37], [38]. Therefore, the compatibility of an implanted biomaterial with the endothelium is critically important for their biomedical applications. As such, we investigated the biocompatibility of a colloidal suspension of Ti_3_C_2_T_x_ MXene nanosheets with human umbilical vein endothelial cells (HUVECs).

**Fig. 3:**
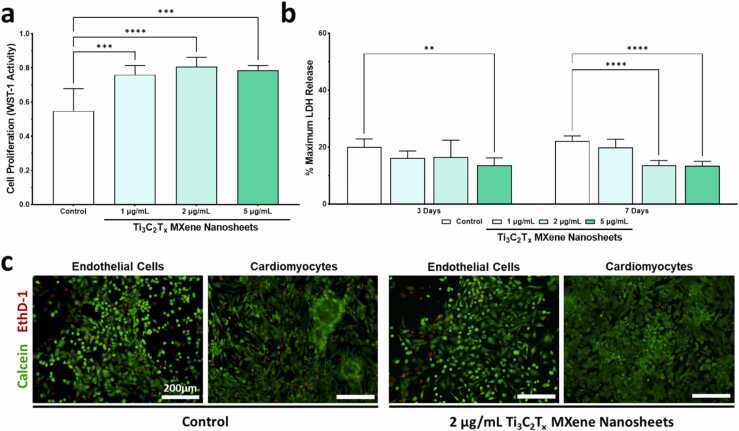
Biocompatibility of Ti_3_C_2_T_x_ MXene nanosheets. The biocompatibility of Ti_3_C_2_T_x_ MXene nanosheets was assessed through culture with human umbilical vein endothelial cells (HUVECs). **a** Proliferation of HUVECs in cultures with different concentrations of Ti_3_C_2_T_x_ MXene nanosheets was assessed using a WST-1 activity assay. After 7 days, increased proliferation of HUVECs was observed when cultured with 1, 2, and 5 µg/mL of Ti_3_C_2_T_x_ MXene nanosheets. Five biological replicates were included per group. **b** Cytotoxicity of Ti_3_C_2_T_x_ MXene nanosheets to HUVECs was assessed at 3 and 7 days using a LDH release assay. No significant increase in LDH release was observed at any of the tested concentrations. Six biological replicates were included per group. **c** Representative live (Calcein) / dead (EthD-1) images were taken after 24 h in cultures of HUVECs and iPSC-derived cardiomyocytes with 2 µg/mL of Ti_3_C_2_T_x_ MXene nanosheets. Cells exhibited excellent biocompatibility with no significant changes in morphology observed when compared to control samples.

The Ti_3_C_2_T_x_ MXene nanosheets at concentrations of 1, 2, and 5 μg/mL were cultured with HUVECs and cell viability as well as proliferation were determined after 7 days of culture. Our data demonstrate that Ti_3_C_2_T_x_ MXene was highly biocompatible with HUVECs. At all three doses tested, the Ti_3_C_2_T_x_ MXene appeared to increase the growth and proliferation of cultured HUVECs (Fig. 3a). To corroborate these findings, the lactate dehydrogenase (LDH) release assay was used at 3 and 7 days of culture to directly measure the cytotoxicity of Ti_3_C_2_T_x_ MXene nanosheets toward HUVECs. There was no significant increase observed in LDH release at all three concentrations of Ti_3_C_2_T_x_ MXene in HUVECS after 3 or 7 days of culture (Fig. 3b) [39]. In fact, Ti_3_C_2_T_x_ MXene did not exert obvious cytotoxicity to HUVECs at doses up to 40 μg/mL, though we observed a decrease in proliferation of HUVECs at doses beyond 20 μg/mL (Supplementary Fig. S2). These findings are in line with what has been previously reported on the toxicity profile of Ti_3_C_2_T_x_ MXene (Supplementary Table S5). Based on these data, we selected a concentration of 2 μg/mL for subsequent studies, and at this dose Ti_3_C_2_T_x_ MXene was highly biocompatible with HUVECs and iPSC-derived cardiomyocytes (iPSC-CM) (Fig. 3c). We did not observe any changes in morphology of both these cell types after 24 h of treatment at 2 μg/mL.

### Ti_3_C_2_T_x_ MXene nanosheets downregulated inflammation-associated surface molecules in HUVECs

Next, we evaluated the molecular effects of Ti_3_C_2_T_x_ MXene nanosheets on HUVECs using targeted quantitative PCR. In the early stages of cardiac allograft vasculopathy, a sequence of events including endothelial presentation of donor antigens through human leukocyte antigen (HLA) I and II, secretion of chemokines, expression of surface adhesion molecules, and expression of lymphocyte co-stimulatory signals contribute synergistically to recipient immune system activation [40], [41], [42]. We have previously demonstrated that Ti_3_C_2_T_x_ MXene quantum dots can exert anti-inflammatory effects at doses as low as 2 μg/mL [43]. Furthermore, others have since reported that Ti_3_C_2_T_x_ MXene quantum dots at 6.25 μg/mL significantly reduce endothelial secretion of the pro-inflammatory cytokines IL-6 and IL-8 [39]. Therefore, direct activity of Ti_3_C_2_T_x_ MXene on the vascular endothelium may have potential to blunt the pathogenesis of allograft vasculopathy.

**Fig. 4:**
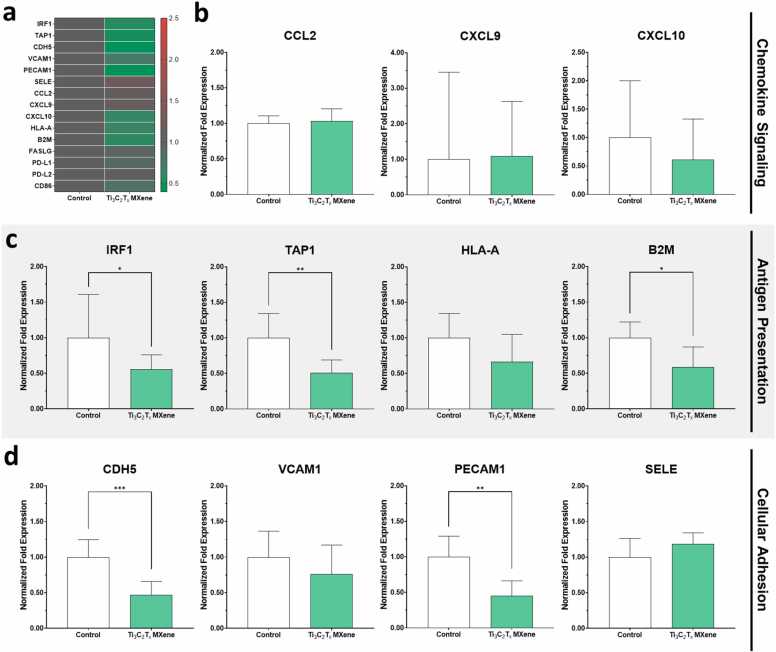
Effects of Ti_3_C_2_T_x_ MXene nanosheets on endothelial cells. The effects of Ti_3_C_2_T_x_ MXene nanosheets at a concentration of 2 µg/mL on chemokine signaling, antigen presentation, cell adhesion, and immune activation within human umbilical vein endothelial cells (HUVECs) was assessed using quantitative PCR. **a** Heat map showing expression of 15 different genes within different treatment conditions. Generally, treatment with 2 µg/mL Ti_3_C_2_T_x_ MXene nanosheets reduced the expression of genes related to antigen presentation and cellular adhesion. **b-d** In particular, culture with of Ti_3_C_2_T_x_ MXene nanosheets decreased the expression of IRF1, TAP1, and B2M, which are all involved in antigen presentation within human leukocyte antigen (HLA) class I. Six biological replicates were included per treatment group.

In the current study, we found that treatment of HUVECs with 2 μg/mL of Ti_3_C_2_T_x_ MXene nanosheets for 48 h significantly reduced endothelial expression of several key proteins involved in antigen presentation and cellular adhesion (Fig. 4a to 4d, Supplementary Fig. S3). In fact, the HUVECs exhibited a unique protective and immunomodulatory phenotype after treatment with MXene nanosheets. In particular, as shown in Fig. 4c, significant downregulation was observed in several key mediators of antigen presentation by HLA Class I, including interferon regulatory factor 1 (IRF1), transporter associated with antigen processing 1 (TAP1), and beta-2 microglobulin (B2M). Interestingly, we also observed a decrease in expression of the cell adhesion molecules including platelet endothelial cell adhesion molecule precursor 1 (PECAM1) and vascular endothelial (VE)-cadherin (CDH5) amongst HUVECs treated with Ti_3_C_2_T_x_ MXene nanosheets (Fig. 4d). However, the HUVECs after treatment with MXene nanosheets maintained normal endothelial phenotype and function, including their capacity for cytokine-induced upregulation of leukocyte adhesion molecules after treatment with interferon-γ (IFN-γ) for 24 h (Supplementary Fig. S4). Nevertheless, this change may contribute to blunting of leukocyte trafficking in the early stages of inflammation [44]. In fact, treatment of these activated HUVECs with Ti_3_C_2_T_x_ MXene nanosheets partially attenuated pro-inflammatory phenotype triggered by IFN-γ (Supplementary Fig. S5).

### Reduction of endothelial-dependent activation of allogeneic T-lymphocytes by Ti_3_C_2_T_x_ MXene nanosheets

Next, we assessed the capacity of Ti_3_C_2_T_x_ MXene nanosheets to reduce allogeneic recognition of endothelial cells by human peripheral blood T-lymphocytes. During cardiac transplantation, unavoidable ischemia-reperfusion injury triggers varying degrees of endothelial dysfunction, resulting in upregulation of both HLA class I and class II in response. These cell surface molecules then facilitate activation and recruitment of alloreactive T-lymphocytes to transplanted hearts. Of note, HLA class I mediated activation of CD8^+^ T-lymphocytes results in lymphocyte proliferation, secretion of pro-inflammatory cytokines, and expression of Granzyme B to cause further allograft injury, triggering the acute phase of cell-mediated rejection [45]. On the other hand, upregulation of HLA class II results in the activation of CD4^+^ T-lymphocytes, polarizing them towards a pro-inflammatory type 1 T-helper (T_H_1) phenotype. This induces the secretion of large amounts of pro-inflammatory cytokines that trigger a cascade of downstream signalling pathways, resulting in chronic cell-mediated rejection, inflammation, and smooth muscle cell proliferation within the vessel wall of the allograft [38].

**Fig. 5:**
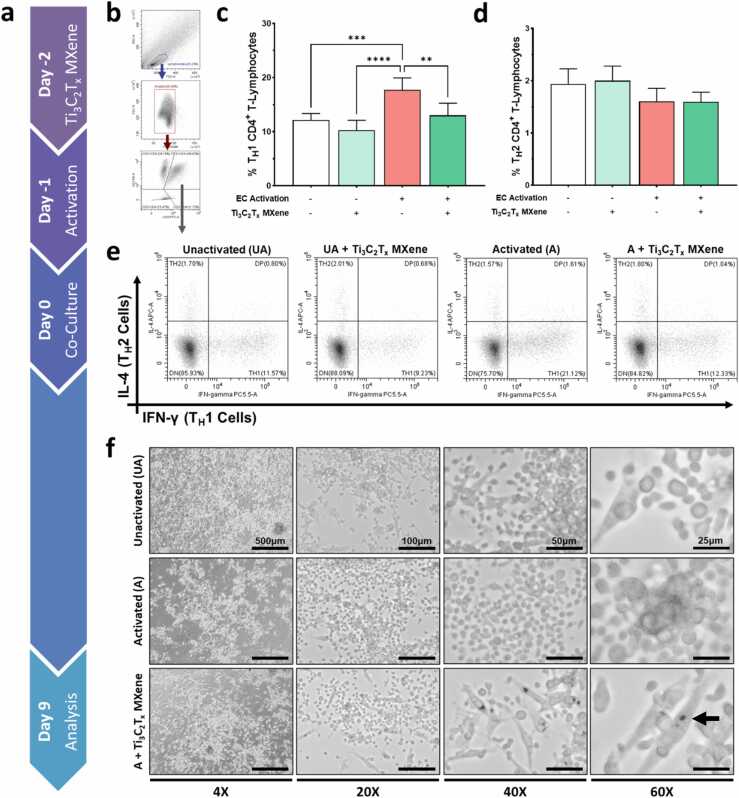
Immunomodulatory effects of Ti_3_C_2_T_x_ MXene nanosheets. The immunomodulatory effects of Ti_3_C_2_T_x_ MXene nanosheets were tested using an *in vitro* model of allogeneic transplant rejection. **a** Briefly, human umbilical vein endothelial cells (HUVECs) were treated with 2 µg/mL of Ti_3_C_2_T_x_ MXene nanosheets for 24 h, and then activated using interferon-γ for 24 h. The cells were then co-cultured with human peripheral blood mononuclear cells (PBMNCs). After 9 days of co-culture, the PBMNCs in suspension were collected and analyzed using flow cytometry. **b** The gating strategy for flow cytometry is shown here. The T-cell population is represented by lymphocytes double positive for both CD3^+^ and CD4^+^. **c-e** Treatment with Ti_3_C_2_T_x_ MXene nanosheets significantly reduced the ability of activated endothelial cells to polarize lymphocytes into a T_H_1 phenotype. These changes occurred without significant increase to the population of T_H_2 lymphocytes. **f** These findings were further corroborated by bright-field microscopy images taken on day 7 of co-culture. When compared to the activated control, there was obvious internalization of Ti_3_C_2_T_x_ MXene nanosheets in endothelial cells (arrow). Additionally, there appeared to be decreased lymphocyte activation (clumping) and endothelial survival within the Ti_3_C_2_T_x_ MXene treated group. Six biological replicates were included per group.

In the current study, HUVECs were treated with 2 μg/mL of Ti_3_C_2_T_x_ MXene nanosheets and subsequently activated using 10 units/mL of IFN-γ (Fig. 5a). IFN-γ is a potent inducer of endothelial antigen presentation and leukocyte recruitment (Supplementary Fig. S6). Next, allogeneic human peripheral blood mononuclear cells (PBMNCs) were added to the cell cultures for 9 days, after which the CD3^+^CD4^+^ T-helper populations were phenotyped using flow cytometry (Fig. 5b to 5e, Supplementary Fig. S7). We found that Ti_3_C_2_T_x_ MXene did not exert any direct adverse effects on the lymphocytes in culture (Supplementary Fig. S8). Amongst PBMNCs co-cultured with activated HUVECs, treatment with 2 μg/mL Ti_3_C_2_T_x_ MXene resulted in a 26% decrease in the percentage of type 1 T-helper (T_H_1) CD4^+^ lymphocytes (Control 17.74%, Ti_3_C_2_T_x_ MXene 13.04%, p < 0.01; Fig. 5c). A numerical trend could also be observed amongst lymphocytes cultured with unactivated HUVECs, though this difference was not statistically significant. Furthermore, this effect appeared to be specific to the T_H_1 population, with no significant differences observed in the type 2 T-helper (T_H_2) populations (Fig. 5d). This targeted suppression of the T_H_1 population has important implications for therapeutic applications of Ti_3_C_2_T_x_ MXene nanosheets. The proportion of T_H_1 cells had been shown to increase in human heart transplant patients over time and this correlated with the development of cardiac allograft vasculopathy[46]. Targeting T_H_1 signaling pathways through IFN-γ depletion or knockdown of STAT proteins has also been shown to dramatically reduce the development of allograft vasculopathy in murine models[47], [48].

In addition, we examined the co-cultured cells using bright-field microscopy after 7 days of culture (Fig. 5f). Notably, Ti_3_C_2_T_x_ MXene nanosheets were selectively uptaken into endothelial cells (and not lymphocytes) in our co-culture experiments. Endothelial uptake of Ti_3_C_2_T_x_ MXene was also confirmed using high resolution fluorescence microscopy, where MXene could be detected based on its autofluorescence (Supplementary Fig. S9). Qualitatively, in the co-culture, there appeared to be decreased lymphocyte clumping and increased endothelial survival within the Ti_3_C_2_T_x_ MXene group. These differences are likely attributed to higher levels of CD8^+^ T-cell activation within the control group, which may have been acting as cytotoxic effector cells against the allogeneic endothelial cells. Activation of CD8^+^ T-cells contributes synergistically with CD4^+^ T-cells to the ongoing inflammatory response. Treatment with Ti_3_C_2_T_x_ MXene nanosheets appeared to directly interrupt this allogeneic immune response cascade.

**Fig. 6:**
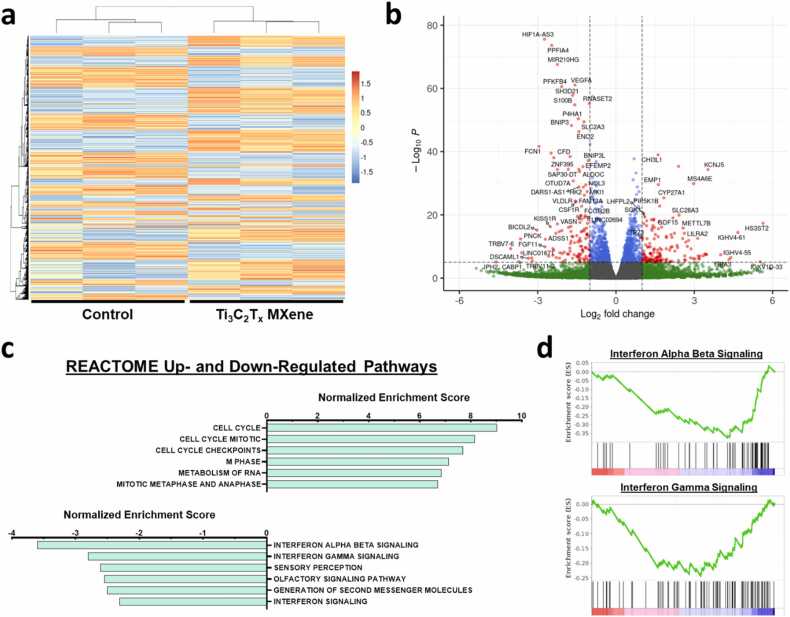
RNA sequencing analysis of lymphocytes co-cultured with Ti_3_C_2_T_x_ MXene-treated endothelial cells. Human peripheral blood mononuclear cells were co-cultured with human umbilical vein endothelial cells (HUVECs) that were treated with Ti_3_C_2_T_x_ MXene nanosheets and activated with interferon-γ. a,b A total of 37,005 genes were identified within lymphocyte samples by RNA sequencing with 2323 genes (6.3%) significantly upregulated and 2137 genes (5.8%) significantly downregulated in lymphocytes co-cultured with HUVECs that were treated using 2 μg/mL of Ti_3_C_2_T_x_ MXene nanosheets. c Gene set enrichment analysis was performed against 1026 REACTOME gene sets using GSEA. The top upregulated pathways by normalized enrichment score were those related to cell cycle regulation, while the top downregulated pathways were related to interferon signaling. d In particular, both interferon alpha beta signaling and interferon gamma signaling had significant enrichment of downregulated genes amongst lymphocytes co-cultured with Ti_3_C_2_T_x_ MXene nanosheets-treated HUVECs. Three biological replicates were included per group.

To understand the depth of these effects, we performed global gene expression analysis of lymphocytes co-cultured with activated HUVECs. Using Illumina RNA sequencing (RNA-Seq), a total of 37,005 genes were identified within the lymphocyte samples, with 2323 genes (6.3%) significantly upregulated and 2137 genes (5.8%) significantly downregulated in PBMNCs co-cultured with Ti_3_C_2_T_x_ MXene-treated HUVECs (Fig. 6a, 6b). Using principal component analysis, the two treatment groups separated into well-defined clusters along the first principal component accounting for 44% of the variance within samples (Supplementary Fig. S10). This was further corroborated by hierarchical clustering using Pearson’s correlation, where intergroup variability greatly exceeding variability within each treatment group (Supplementary Fig. S11).

Next, we performed gene set enrichment analysis (GSEA) in lymphocytes to identify pertinent pathways affected by Ti_3_C_2_T_x_ MXene-treated endothelial cells. Across 1026 REACTOME gene sets used in the analysis, 750 (73.1%) were upregulated and 276 (26.9%) were downregulated in the treatment group, spanning broad biological processes (Supplementary Fig. S12). Amongst these, 392 of the upregulated gene sets (52.3%) and 6 of the downregulated gene sets (2.2%) were significantly enriched at a false discovery rate (FDR)< 0.05. In particular, the top enriched upregulated pathways largely pertained to cell cycle regulation within lymphocytes (Fig. 6c, Supplementary Fig. S13). This included a highly significant increase in the expression of the cyclin-dependent kinase inhibitor p21^Cip1^, which plays a critical role in the inhibition of transplant-induced T-cell activation, cell-mediated rejection, and the development of allograft vasculopathy (Supplementary Fig. S14) [49], [50], [51]. Furthermore, both interferon alpha/beta signaling and interferon gamma were amongst the top enriched downregulated pathways (Fig. 6c, 6d, Supplementary Fig. S15 to S18).

These changes were substantiated by trends signifying decreases in T-cell receptor activation and signalling within the treatment group. Expression levels of the invariant T-cell receptor (TCR) proteins CD3ε (CD3E), CD3ζ (CD247), as well as the key activation-associated tyrosine kinase ZAP-70 were all significantly downregulated within lymphocytes after treatment with Ti_3_C_2_T_x_ MXene, with additional strong downregulatory trends observed in the TCR proteins CD3δ (CD3D), CD3γ (CD3G), and the T-cell co-stimulatory receptor CD28 (Supplementary Fig. S19). These changes appeared to correlate with CD4^+^ T-lymphocytes, as significant downregulation was also observed in the CD4 TCR co-receptor, while no significant differences in expression were noted in the components of cytotoxic CD8^+^ T-lymphocytes (Supplementary Fig. S20). Additionally, strong significant decreases were observed in the expression levels of the macrophage marker CD14, HLA class I and class II, as well as the regulatory protein FOXP3 in the Ti_3_C_2_T_x_ MXene-treated group (Supplementary Fig. S21). The presence of FOXP3 mRNA within transplanted organs, in particular, has been associated with episodes of acute vascular rejection [52], [53]. Taken together, these findings suggest that Ti_3_C_2_T_x_ MXene nanosheets can significantly reduce endothelial immune activation of T-lymphocytes to ameliorate the pathogenesis of allograft vasculopathy.

### Amelioration of transplant vasculopathy *in vivo* in transplanted aortic allografts by Ti_3_C_2_T_x_ MXene nanosheets

**Fig. 7:**
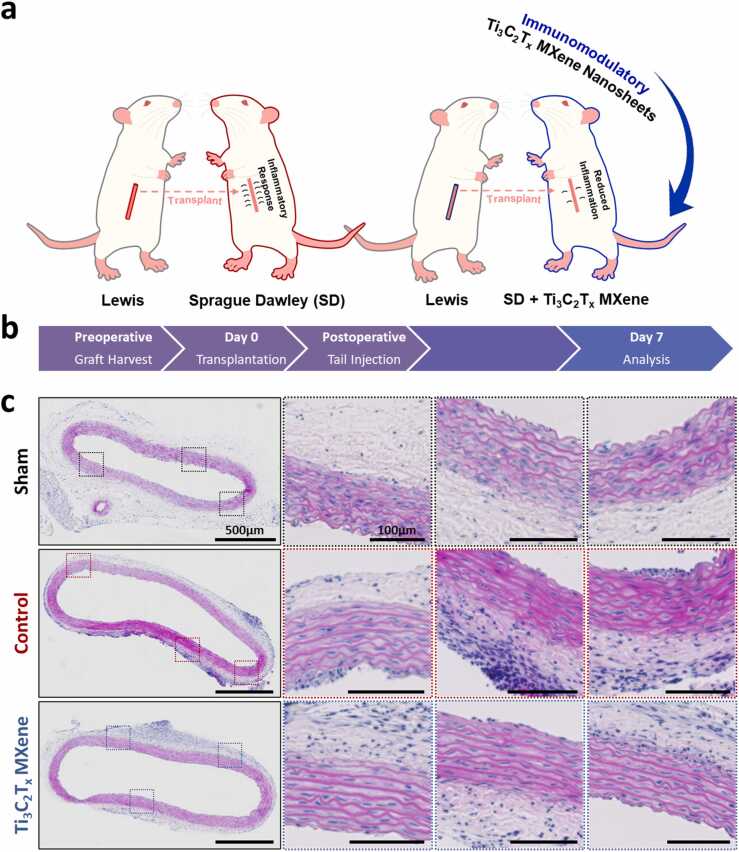
Immunomodulatory effects of Ti_3_C_2_T_x_ MXene nanosheets in an *in vivo* model of cardiac allograft vasculopathy. The *in vivo* immunomodulatory effects of Ti_3_C_2_T_x_ MXene nanosheets using a rat model of allogeneic rejection and allograft vasculopathy. A,b Briefly, segments of the thoracic aorta were harvested from male Lewis rats and transplanted as an interposition graft into the abdominal aorta of male Sprague Dawley (SD) rats. Animals received a 1.5 mg/kg tail vein injection of Ti_3_C_2_T_x_ MXene nanosheets immediately after transplantation. After 7 days, animals were euthanized and tissues were collected for analysis. C On staining with hematoxylin and eosin, transplanted aortic segments from Ti_3_C_2_T_x_ MXene nanosheets-treated animals exhibited less endothelial thickening and adventitial inflammatory infiltration compared to control animals. Three to four animals were included per group.

Next, we assessed the immunomodulatory effects of Ti_3_C_2_T_x_ MXene nanosheets *in vivo* using a rat model of allograft vasculopathy (Fig. 7a). Briefly, an aortic allograft was harvested from the descending thoracic aorta of donor Lewis rats and immediately transplanted as an interposition graft into the infra-renal abdominal aorta of recipient Sprague-Dawley rats. This well-established model mimics the cell-mediated rejection and histopathological changes of allograft vasculopathy typically seen in the coronary circulation of transplanted hearts [54], [55]. Cellular changes, including adventitial infiltration of inflammatory cells and loss of medial integrity, can be visualized as early as one week [56], [57]. Immediately following the operation, recipients received 1.5 mg/kg of Ti_3_C_2_T_x_ MXene nanosheets intravenously through a tail vein injection. Animals were kept for 7 days, after which the allograft and plasma were collected for analysis (Fig. 7b). There were no mortalities or other adverse events reported during the observation period, and no changes were observed in the appearance or behaviour of treated animals. Furthermore, no histologic changes were observed in the liver, kidney, and lungs of animals treated with Ti_3_C_2_T_x_ MXene nanosheets (Supplementary Fig. S22). Nevertheless, clear histopathological differences were observed in the transplant aortic segments between control animals and those treated with Ti_3_C_2_T_x_ MXene nanosheets. In particular, treated animals had fewer regions of adventitial inflammatory infiltration when compared to control animals (Fig. 7**c**). When examined using immunohistochemistry, we observed a strong trend towards fewer cytotoxic CD8^+^ T-lymphocyte infiltration into the adventitia of Ti_3_C_2_T_x_ MXene treated animals (Supplementary Fig. S23). Additionally, quantitative PCR of peripheral blood mononuclear cells from these animals showed significant decreases in the expression of key genes associated with T-lymphocytes, including CD4 and CD28 (Supplementary Fig. S24). Finally, treated animals had fewer regions of endothelial thickening when compared to control animals. These changes were highly suggestive of *in vivo* immunomodulatory effects exerted by Ti_3_C_2_T_x_ MXene nanosheets.

**Fig. 8:**
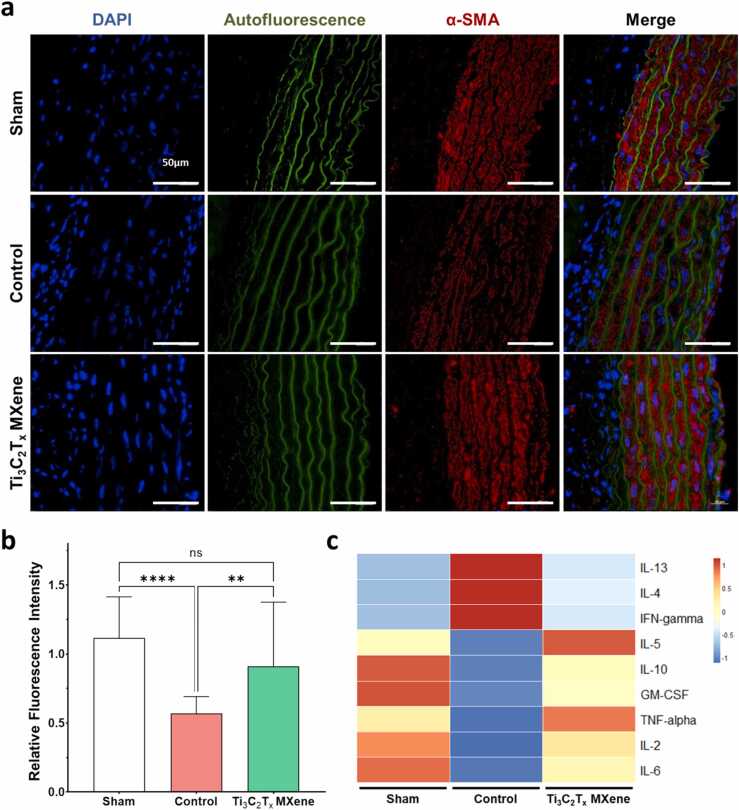
Treatment with Ti_3_C_2_T_x_ MXene nanosheets reduce allograft damage and inflammatory response in a rat model of cardiac allograft vasculopathy. Immunofluorescence staining and a multiplex ELISA were performed to quantify the degree of rejection against the allografts. A,b Immunohistochemistry was performed against alpha-smooth muscle actin (α-SMA) as a marker for integrity of the vessel media. The abdominal aorta segments of each animal were normalized to a segment of the native thoracic aorta of the same animal. There was significantly decreased staining for α-SMA amongst transplanted animals when compared to the sham animals. These changes were ameliorated in animals treated with Ti_3_C_2_T_x_ MXene nanosheets. **C** Multiplex ELISA against several cytokines was performed using blood plasma. As shown here, animals treated with Ti_3_C_2_T_x_ MXene nanosheets had a cytokine profile that resembled those of sham animals. In particular, there were lower levels of the pro-inflammatory cytokine interferon-gamma. Three to four biological replicates were included per group.

To further corroborate these findings, the medial integrity of transplant allografts was assessed using immunohistochemistry for alpha-smooth muscle actin (α-SMA) within the media (Fig. 8a, 8b). The loss of α-SMA expressing medial smooth muscle cells occurs within days after transplantation and is a hallmark sign of early allograft rejection [57], [58], [59]. This change occurs primarily through apoptosis mediated by infiltrating cytotoxic CD8^+^ T-lymphocytes. Since Ti_3_C_2_T_x_ MXene nanosheets reduced CD8^+^ T-lymphocyte infiltration into the allografts, we expected to see improved medial integrity within MXene-treated animals. For each animal, a non-transplanted segment of the descending thoracic aorta was taken as a control for normalization of the fluorescence intensity. As seen here, control animals who have received an allograft had significantly reduced α-SMA fluorescence at one week when compared to surgical shams (Sham 1.116, Control 0.569, p < 0.0001). Treatment with Ti_3_C_2_T_x_ MXene nanosheets, however, seemed to have significantly ameliorated medial damage within the allograft (Control 0.569, Ti_3_C_2_T_x_ MXene 0.910, p < 0.01). These changes were supported by alterations in circulating cytokine levels, where Ti_3_C_2_T_x_ MXene treated animals had lower plasma levels of pro-inflammatory cytokines, and an overall cytokine profile that resembled that of sham animals (Fig. 8**c**). Taken together, these findings are in line with our *in vitro* data and strongly support an *in vivo* role for immunomodulatory Ti_3_C_2_T_x_ MXene nanosheets in the treatment of cardiac allograft vasculopathy.

## Conclusion

In summary, we have reported the first application of immunomodulatory titanium carbide (Ti_3_C_2_T_x_) MXene for treatment of allograft vasculopathy. In particular, Ti_3_C_2_T_x_ MXene nanosheets regulated endothelial antigen presentation and cellular adhesion, which consequently reduced their ability to drive lymphocyte activation, proliferation, and interferon signaling. Furthermore, we demonstrated that treatment with Ti_3_C_2_T_x_ MXene nanosheets reduced immune cell infiltration and allograft injury when applied in an *in vivo* model of cardiac allograft vasculopathy. These findings are promising and support the future development of Ti_3_C_2_T_x_ MXene nanosheets into a platform for prevention of allograft rejection in the heart and other solid organs. This research also paves the path for the advancement of Ti_3_C_2_T_x_ MXene technologies for other immune-sensitive regenerative medicine applications.

In addition to direct immunomodulatory role of Ti_3_C_2_T_x_ MXene nanosheets the current field is moving toward development of different MXenes to be used for synergetic bioactivities for immunoengineered nanosystems. In fact, application of MXene biomaterials as immunomodulatory or immunotherapy agents could offer unique advantages including facile fabrication and suitable physicochemical properties to mimic specific biological activities. One of these major applications is targeted drug delivery of therapeutic interventions such as anti-cancer drug delivery and material-assisted antimicrobial activities. With respect to anti-cancer applications of MXene nanosystems, their intrinsic photothermal and photodynamic properties make them desirable candidates for light-activated and combined chemo-photothermal therapies to treat cancer (Supplementary Table S6 to S8**)**. More recently, it has been reported that the chemical composition, size, and dimension of MXenes affect their biological properties in a significant way. For instance, quantum dots and heterostructures derived from 2D MXene biomaterials exhibited advantages of enhanced biocompatibility, drug loading capacity and pH-responsive drug release properties and photothermal conversion efficiency (Supplementary Table S6 to S8**)**. These improvements could be associated with increased active surface area, dispersibility, and durability of these materials when interacting with biological environments. Together, these advantages support the potential of MXene for future translational applications and combined therapies. However, their application for the targeted approaches is currently at early stages; and further investigations are required to fully realize and confirm their capabilities for immunotherapy, immunoengineering, and regenerative medicine-related applications.

## Materials and methods

### Preparation of colloidal suspension of Ti_3_C_2_T_x_ MXene nanosheets

The Ti_3_C_2_T_x_ MXene nanosheets were obtained from commercial source (Laizhou Kai Kai Ceramic Materials Co., Ltd, China). Ti_3_C_2_T_x_ nanosheets can be synthesized from MAX phase using either top-down or bottom-up approach (Supplementary Fig. S25). To prepare colloidal suspensions, Ti_3_C_2_T_x_ flakes were dispersed in saline solution (0.9% NaCl) and followed by sonication treatment in bath ultrasonic for 60 min. The obtained colloidal suspension contained mono-, few-, and multi-layers of Ti_3_C_2_T_x,_ nanosheets and was stored at 4 °C for further experiments.

### Physicochemical characterization

The microstructural properties of the material were characterized using scanning electron microscopy (FEI Nova, NanoSEM 450 ThermoFisher Scientific Co.), transmission electron microscopy (FEI Talos, F200X S/TEM, ThermoFisher Scientific Co.) at the Manitoba Institute of Materials (MIM), University of Manitoba, Canada. X-ray photoelectron spectroscopy measurement was performed on Ti_3_C_2_T_x_ nanosheets using PHI Quantera XPS at the Rice University, United States. The X-ray diffraction (XRD) analysis of material was performed using the Rigaku XRD Ultima IV at University of Alberta, Canada at 2-theta 5–80° using a continuous scan with speed rate of 2.000°/min.

### Animals

All animal protocols conform to the standards and guidelines set out by the Canadian Council on Animal Care and were approved by the University of Manitoba Animal Care Committee (AC11467) prior to commencement of studies. Male Lewis rats weighing 260–280 g were obtained from Charles River Laboratories and used as donors. Male Sprague-Dawley rats weighing 260–280 g were obtained from Central Animal Care Services at the University of Manitoba and used as recipients.

### Cell culture

Human umbilical vein endothelial cells were obtained from Lonza (C2519A) and cultured in EGM-2 medium (CC-3162, Lonza) using manufacturer protocols unless otherwise specified. Briefly, cryopreserved cells were thawed in pre-warmed EGM-2 and seeded at a density of 2500 cells/cm^2^. EGM-2 was changed 24 h after seeding and every 2 days thereafter. Cells were sub-cultured using Trypsin (25200056, Gibco) at 80% confluency. Cells from passages 3–5 were used for experiments.

Human peripheral blood mononuclear cells (PBMNCs) were isolated from whole blood obtained from healthy volunteer donors. All procedures were approved by the University of Manitoba Biomedical Research Ethics Board (HS18974). The PBMNCs were isolated using Lympholyte®-H Cell Separation Media (CL5015, Cedarlane Labs). For isolated cell cultures, PBMNCs were activated with plate-bound anti-CD3 antibody (300313, BioLegend) and 2 μg/mL of soluble anti-CD28 antibody (302913, BioLegend) immediately after isolation, and then cultured in Advanced RPMI 1640 medium (12633012, Gibco) supplemented with 10% FBS (12483020, Gibco), 2 mM GlutaMAX (35050061, Gibco), 1:100 Penicillin-Streptomycin (15140122, Gibco), 0.055 mM 2-mercaptoethanol (M3148, Sigma-Aldrich) and 20 units/mL recombinant human IL-2 (589102, BioLegend). To induce T_H_1 polarization, 10 ng/mL of recombinant human IL-12 (573002, BioLegend) was added to the culture medium. The co-cultures of HUVECs and human PBMNCs were performed using freshly isolated PBMNCs and HUVECs in EGM-2 medium supplemented with 20 units/mL of recombinant human IL-2.

### Cell proliferation assay

Proliferation of HUVECs was assessed using WST-1 Cell Proliferation Assay kit (K304, BioVision Inc.). Briefly, HUVECs were grown to 80% confluency in 96 well plates and then treated with Ti_3_C_2_T_x_ MXene nanosheets at different concentrations 1, 2 and 5 µg/mL. After 7 days, cells were treated with WST-1 reagent and absorbance was quantified using the Cytation 5 Imaging Multi-Mode Reader (BioTek) at 450 nm (reference wavelength 650 nm). Five biological replicates were included for each treatment condition.

### Lactate dehydrogenase release assay

The cytotoxicity to HUVECs after treatment with Ti_3_C_2_T_x_ MXene nanosheets was assessed using Lactate Dehydrogenase (LDH) Cytotoxicity Detection Kit (MK401, Takara Bio). Briefly, HUVECs were grown to 80% confluency and then treated with Ti_3_C_2_T_x_ MXene at 1, 2 and 5 µg/mL for 7 days. Medium was taken from culture wells at 3 and 7 days for assessment of LDH release. Absorbance was measured at 492 nm (with a reference wavelength of 620 nm) using the Cytation5 Imaging Multi-Mode Reader (BioTek). Six biological replicates were included for each treatment condition.

### Quantitative polymerase chain reaction (qPCR)

HUVECs were grown to 80% confluency and treated with Ti_3_C_2_T_x_ MXene nanosheets at 2 µg/mL for 48 h. Total cellular RNA was isolated using the Aurum™ Total RNA Mini Kit (7326820, Bio-Rad) and quantified using a NanoDrop™ Spectrophotometer (Thermo Fisher Scientific). cDNA was synthesized using the High-Capacity cDNA Reverse Transcriptase Kit (4368814, Thermo Fisher Scientific). qPCR was performed on the CFX384 Touch Real-Time PCR Detection System (Bio-Rad) and gene expression was quantified using the 2^-ΔΔCt^ method, using beta-actin (ACTB) and GAPDH as endogenous controls [60]. Six biological replicates were included for each treatment condition. A list of the primers used for qPCR analysis is presented in Supplementary Table S9.

### Western blotting

Activation of HUVECs in the presence and absence of Ti_3_C_2_T_x_ MXene nanosheets was assessed using western blot analysis for HLA class II (HLA-DRα) and ICAM-1. Briefly, HUVECs at 80% confluency were pre-treated with 2 µg/mL of Ti_3_C_2_T_x_ MXene for 24 h. The cells were then activated using 10 units/mL of recombinant human IFN-γ (570202, BioLegend) for 24 h and collected in RIPA buffer on ice and stored at 80 ºC for 24 h. After thawing, cell lysate was spun at 12,000 g for 10 min to collect the supernatant. Protein was quantified using the Bradford Protein Quantification Assay (5000006, Bio-Rad). For gel electrophoresis, 30 µg of protein was loaded into a 10% polyacrylamide resolving gel (1610658, Bio-Rad) and run at 100 V, and then transferred onto a PVDF membrane. The desired primary antibody was incubated with the membrane overnight at 4 °C. The secondary antibodies were added for 1 h at room temperature. Signal was detected using the Pierce™ ECL Western Blotting Substrate (32209, Thermo Fisher Scientific) and a ChemiDoc MP (Bio-Rad). Signal intensities were quantified using Quantity One 1-D Analysis Software (Bio-Rad, Hercules, California) and normalized to beta-actin. Four biological replicates were included for each treatment condition. The list of primary and secondary antibodies used for probing the membranes, along with their dilutions, is presented in Supplementary Table S10.

### Immunomodulation assay and flow cytometry

Flow cytometry was used to characterize the immunomodulatory effects of Ti_3_C_2_T_x_ MXene nanosheets. Briefly, HUVECs at 80% confluency were pre-treated with 2 μg/mL of Ti_3_C_2_T_x_ MXene for 24 h, after which 10 units/mL of IFN-γ was added to the cell culture for 24 h. Subsequently, the media was replaced with EGM-2 supplemented with 20 units/mL of IL-2 and freshly isolated human PBMNCs were added to the co-culture. Ti_3_C_2_T_x_ MXene at 2 μg/mL was also included in the media for the treatment groups. Co-cultures were maintained for 9 days, during which light microscopy images were captured using the Cytation5 Imaging Multi-Mode Reader. After 9 days, cells were pulsed with the Cell Stimulation Cocktail with protein transport inhibitor (00–4975–93, Thermo Fisher Scientific) for 6 h and collected in ice-cold FACS buffer (containing 1% bovine serum albumin, 2 mM EDTA, and 0.1% sodium azide) for analysis. For this, cells were then fixed and permeabilized using the eBioscience™ Staining Buffer Set (00–5523–00, Thermo Fisher Scientific) and stained for 1 h at room temperature using manufacturer recommended antibody concentrations. Prior to analysis, cells were washed once and resuspended in up to 100 µL with FACS buffer. Cells were analyzed on the CytoFLEX Flow Cytometer (Beckman Coulter) with the appropriate isotype and gating controls. Data analysis was performed using CytExpert Software version 2.3.1.22 (Beckman Coulter, Brea, California). The list of antibodies used for flow cytometry and their concentrations is presented in Supplementary Table S11.

### Transcriptome sequencing

HUVECs at 80% confluency were pre-treated with 2 μg/mL of Ti_3_C_2_T_x_ MXene for 24 h, after which 10 units/mL of IFN-γ was added to the cell culture for 24 h. Subsequently, the media was replaced with EGM-2 supplemented with 20 units/mL of IL-2 and freshly isolated human PBMNCs were added to the co-culture. Ti_3_C_2_T_x_ MXene at 2 μg/mL was also included in the media for the treatment groups. PBMNCs were collected after 9 days, from which total RNA was isolated using the Aurum™ Total RNA Mini Kit (7326820, Bio-Rad). Bioanalyzer quality assessment, library construction, and Illumina RNA sequencing was conducted at Génome Quebec CES (Montréal, Québec, Canada). A minimum of 50 million 100 bp paired-end reads were collected per sample using the Illumina NovaSeq 6000 S4 platform (San Diego, California). Three biological replicates were run for each treatment condition.

Quality control of raw and processed FASTQ sequence files was performed using FastQC v0.11.9 [61]. Illumina error correction was performed using Rcorrector v1.0.4 and TranscriptomeAssemblyTools from Harvard Informatics [62], [63]. Adapter trimming was performed using Trim Galore! V0.6.5 [64]. Genome alignment was performed using STAR v2.7.9a and confirmed with pseudoalignments using kallisto v0.461[65], [66]. The human genome GRCh38.p13 Ensembl release 104 was used for the reference genome and annotations [67]. Quality control of alignments was performed using RNA-SeQC v2.4.2 and Samtools v1.13 was used to sort the alignments [68], [69]. Gene features were counted using featureCounts v2.0.3 and analyzed using DESeq2 v1.32.0 (R v4.1.0 for Debian Linux) [70], [71], [72]. Gene set enrichment analysis (GSEA) was performed using GSEA software v4.1.0 for Linux and MsigDB v7.4 [73], [74], [75]. Pathway visualization was performed using REACTOME v78 [76], [77], [78], [79]. Heatmaps were generated using package NMF v0.23.0 for R and Prism version 9.02 for Windows (GraphPad, San Diego, California) [80]. Volcano plots were generated using package EnhancedVolcano for R v1.10.0 [81].

### *In vivo* allograft vasculopathy model

All animal care and surgical procedures were performed at the R.O. Burrell Laboratory, St. Boniface Hospital Albrechtson Research Centre, University of Manitoba in accordance with standard operating procedures. After induction of anesthesia, donor Lewis rats underwent a median sternotomy where the heart, lungs, and other mediastinal structures were removed. Immediately afterwards, the descending thoracic aorta was carefully mobilized and harvested. The donor aorta was then stored in ice-cold saline for subsequent transplantation. After that recipient Sprague-Dawley rats were placed under general anesthesia, and a median laparotomy was performed. The infrarenal abdominal aorta was carefully dissected out and branch vessels inferior to the gonadal arteries were suture ligated. Next, infrarenal clamps were placed on the abdominal aorta and a 5 mm segment was resected. The previously harvested donor thoracic aortic segment was then trimmed and anastomosed to the abdominal aorta as an interposition graft in a non-reversed fashion using 8–0 Prolene sutures. Hemostasis was ensured using a combination of pressure and SurgiCel. Good pulses were felt at the aortic bifurcation prior to closure. The abdominal viscera were then replaced into the abdomen and the abdomen was closed. Animals were kept for one week, after which the transplanted aortic segment (and a segment of the descending thoracic aorta) was harvested from each animal for subsequent analysis. Blood and plasma was also collected for subsequent analysis.

### Immunohistochemistry

After collection, aortic segments were immediately fixed overnight in 10% buffered formalin (SF100, Fisher Scientific) at room temperature. They were then embedded in paraffin blocks, cut to 5 µm thick sections, and mounted on glass slides. Hematoxylin and eosin staining was performed in a regressive fashion with Harris’ hematoxylin. For immunohistochemistry, sections were rehydrated using ethanol and then incubated with the desired primary antibody overnight at 4 °C. Slides were then washed and incubated with secondary antibody for 1 h at room temperature. Finally, sections were mounted with Prolong™ Diamond Antifade Mountant with DAPI (P36971, Thermo Fisher Scientific) and imaged using a Nikon Ti-2E fluorescence microscope. Six images were taken per section for α-SMA staining, and fluorescence intensity was quantified using Fiji v1.53c [82]. The list of antibodies used, and their respective dilutions, is presented in Supplementary Table S12.

### Multiplex cytokine level quantification

Whole blood was collected from each animal through direct puncture of the left ventricular apex using BD Vacutainer® blood collection tube with K_2_EDTA (367861, BD Life Sciences). These tubes were kept on ice and spun at 1000 g for 10 min at 4 °C to remove cells. The supernatant was further spun at 2000 g for 15 min at 4 °C to remove any platelets, and the resultant plasma was tested for cytokine levels using the LEGENDplex™ Rat Th1/Th2 (9-plex) Panel (740405, BioLegend) bead-based immunoassay. Data was collected on the CytoFLEX Flow Cytometer (Beckman Coulter) and analyzed using the LEGENDplex™ Data Analysis Software version 8.0 (VigeneTech Inc., Carlisle, Massachusetts).

### Quantitative PCR of *in vivo* PBMNCs

PBMNCs were isolated from whole blood using a Ficoll gradient (10831, Sigma-Aldrich). Quantitative PCR of *in vivo* PBMNCs were performed using similar techniques as described above. Two technical replicates were included for each animal. Transferrin receptor (TFRC) and GAPDH were used as endogenous controls. A list of the primers used can be found in Supplementary Table S13**.**

### Statistical analysis

Comparisons of two independent groups were performed using the unpaired two-tailed Students’ t-test. Comparison between three or more independent groups was performed using the one-way analysis of variance (ANOVA) and Tukey’s honestly significant difference test. Comparisons involving two independent variables were performed using a two-way analysis of variance and the Bonferroni multiple comparisons test. An adjusted p-value less than 0.05 was considered to be significant. All statistical analysis was performed using Prism version 9.02 for Windows (GraphPad, San Diego, California).

## CRediT authorship contribution statement

**Weiang Yan:** Conceptualization, Data curation, Formal analysis, Investigation, Methodology, Software, Writing – original draft. **Alireza Rafieerad:** Conceptualization, Data curation, Formal analysis, Investigation, Methodology, Writing – original draft. **Keshav Narayan Alagarsamy:** Data curation, Formal analysis, Investigation, Methodology. **Leena Regi Saleth:** Data curation, Formal analysis, Investigation, Methodology. **Rakesh C. Arora:** Conceptualization, Formal analysis, Methodology, Project administration, Supervision, Validation, Writing – review & editing. **Sanjiv Dhingra:** Conceptualization, Data curation, Formal analysis, Funding acquisition, Investigation, Methodology, Project administration, Resources, Software, Supervision, Validation, Writing – review & editing. **Weiang Yan** and **Alireza Rafieerad** contributed equally to this work.

## Declaration of Competing Interest

The authors declare that they have no known competing financial interests or personal relationships that could have appeared to influence the work reported in this paper.

## Data Availability

The raw and processed data required to reproduce these findings are available from the corresponding authors upon reasonable request.
