# Supplementary material for: Immunoengineered MXene nanosystem for mitigation of alloantigen presentation and prevention of transplant vasculopathy
Source: Nano Today. 2023 Feb;48:None. doi: 10.1016/j.nantod.2022.101706 (PMC10181944; doi:10.1016/j.nantod.2022.101706)
Supplement: Supplementary file 1 — Supplementary material [file mmc1.docx]

**Electronic Supplementary Information**

**Immunoengineered MXene Nanosystem for Mitigation of Alloantigen Presentation and Prevention of Transplant Vasculopathy**

**Weiang Yan^a,b,#^, Alireza Rafieerad^a#^, Keshav Narayan Alagarsamy^a^, Leena Regi Saleth^a^,**  **Rakesh C. Arora^a,b^, Sanjiv Dhingra^a,*^**

*^a^Institute of Cardiovascular Sciences, St. Boniface Hospital Albrechtsen Research Centre, Department of Physiology and Pathophysiology, Max Rady College of Medicine, Rady Faculty of Health Sciences, University of Manitoba, Winnipeg, Manitoba, R2H 2A6, Canada*

*^b^Section of Cardiac Surgery, Department of Surgery, Max Rady College of Medicine, Rady Faculty of Health Sciences, University of Manitoba, Winnipeg, Manitoba, R3E 0W2, Canada*

^#^ Weiang Yan and Alireza Rafieerad contributed equally to this work.

***Correspondence:**

**Sanjiv Dhingra, PhD, FAHA, FAPS**

Institute of Cardiovascular Sciences

St. Boniface Hospital Research Centre

Department of Physiology and Pathophysiology

Rady Faculty of Health Sciences, University of Manitoba

R-3028-2, 351 Tache Avenue, Winnipeg, R2H2A6, Canada

Email: [sdhingra@sbrc.ca](mailto:sdhingra@sbrc.ca)

**List of Supplementary Materials**

1. **Supplementary Figure S1**

Structural and elemental characterization of Ti_3_C_2_T_x_ MXene nanosheets

1. **Supplementary Figure S2**

Effect of Ti_3_C_2_T_x_ MXene nanosheets on endothelial cells at higher concentrations

1. **Supplementary Figure S3**

Endothelial expression of immune system co-activation or co-inhibition signals

1. **Supplementary Figure S4**

Cytokine-induced upregulation of VCAM-1 in endothelial cells

1. **Supplementary Figure S5**

Effects of 2 µg/mL Ti_3_C_2_T_x_ MXene nanosheets on endothelial cells treated with interferon-γ

1. **Supplementary Figure S6**

Interferon-γ induced upregulation of endothelial antigen presentation and leukocyte adhesion

1. **Supplementary Figure S7**

Isotype controls for flow cytometry

1. **Supplementary Figure S8**

Viability of lymphocytes in co-culture with Ti_3_C_2_T_x_ MXene nanosheets

1. **Supplementary Figure S9**

Endothelial uptake of Ti_3_C_2_T_x_ MXene nanosheets

1. **Supplementary Figure S10**

Principal component analysis from RNA sequencing of lymphocytes co-cultured with Ti_3_C_2_T_x_ MXene-treated endothelial cells

1. **Supplementary Figure S11**

Cluster dendrogram of individual RNA sequencing samples

1. **Supplementary Figure S12**

Gene set enrichment analysis of RNA sequencing samples

1. **Supplementary Figure S13**

Gene expression changes in cell cycle checkpoints within lymphocytes co-cultured with Ti_3_C_2_T_x_ MXene-treated endothelial cells, based on RNA sequencing data

1. **Supplementary Figure S14**

Gene expression changes in p21^Cip1^

1. **Supplementary Figure S15**

Gene expression changes in interferon alpha/beta signaling within lymphocytes co-cultured with Ti_3_C_2_T_x_ MXene-treated endothelial cells, based on RNA sequencing data

1. **Supplementary Figure S16**

REACTOME pathway for interferon alpha/beta signaling

1. **Supplementary Figure S17**

Gene expression changes in interferon gamma signaling within lymphocytes co-cultured with Ti_3_C_2_T_x_ MXene-treated endothelial cells, based on RNA sequencing data

1. **Supplementary Figure S18**

REACTOME pathway for interferon gamma signaling

1. **Supplementary Figure S19**

Downregulation of the T-cell receptor, co-stimulator, and its associated tyrosine kinase within lymphocytes co-cultured with Ti_3_C_2_T_x_ MXene-treated endothelial cells, based on RNA sequencing data

1. **Supplementary Figure S20**

Expression of the CD4 and CD8 T-cell co-receptors, as well as key CD8^+^ T-cell associated cytotoxins, based on RNA sequencing data

1. **Supplementary Figure S21**

Expression of macrophage markers, HLA class I and II, and FOXP3, based on RNA sequencing data

1. **Supplementary Figure S22**

H&E staining of lungs, livers, and kidneys from rats after tail vein injection of Ti_3_C_2_T_x_ MXene nanosheets

1. **Supplementary Figure S23**

CD4^+^ and CD8^+^ lymphocyte infiltration in the adventitia of transplanted aortic allografts

1. **Supplementary Figure S24**

Quantitative PCR of peripheral blood mononuclear cells from *in vivo* experiments

1. **Supplementary Figure S25**

Top-down and bottom-up approaches for synthesis of 2D MXenes

1. **Supplementary Table S1**

The XPS peak identification of Ti 2p in Ti_3_C_2_T_x_ MXene nanosheets

1. **Supplementary Table S2**

The XPS peak identification of C 1s in Ti_3_C_2_T_x_ MXene nanosheets

1. **Supplementary Table S3**

The XPS peak identification of O 1s in Ti_3_C_2_T_x_ MXene nanosheets

1. **Supplementary Table S4**

The XPS peak identification of F 1s in Ti_3_C_2_T_x_ MXene nanosheets

1. **Supplementary Table S5**

Biocompatibility of various MXene compositions

1. **Supplementary Table S6**

Applications of different types of MXene based nanosystems for immunologic applications

1. **Supplementary Table S7**

Some applications of MXene-based immunoengineered nanosystems for combination therapy

1. **Supplementary Table S8**

Application of MXene based nanosystems for photo-thermal therapy applications

1. **Supplementary Table S9**

List of human quantitative PCR primers used in this study

1. **Supplementary Table S10**

List of western blotting antibodies used in this study

1. **Supplementary Table S11**

List of flow cytometry antibodies used in this study

1. **Supplementary Table S12**

List of immunocytochemistry antibodies used in this study

1. **Supplementary Table S13**

List of rat quantitative PCR primers used in this study


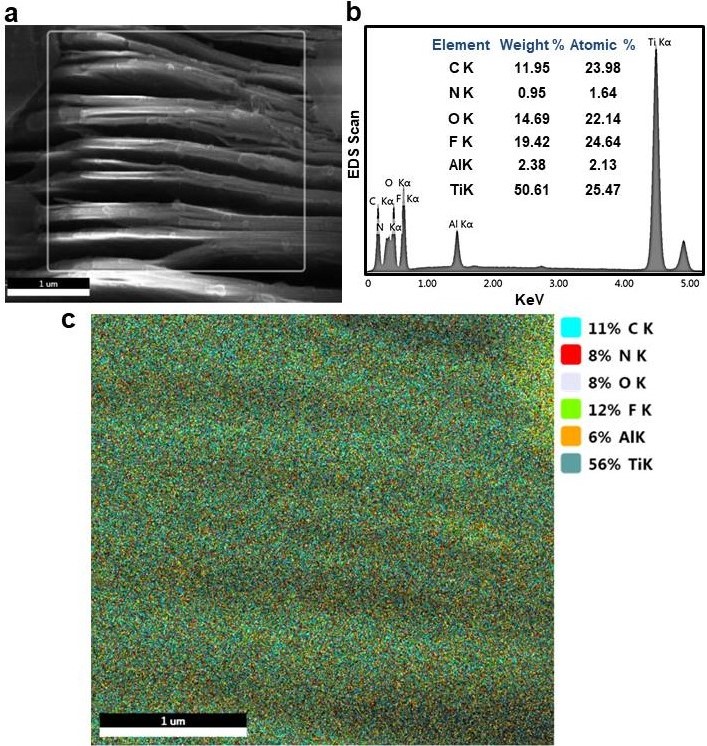


**Supplementary Figure S1. Structural and elemental characterization of Ti_3_C_2_T_x_ MXene nanosheets.** **a**,**b** SEM image and its corresponding EDS scan analysis of 2D Ti_3_C_2_T_x_ MXene. The morphology of material revealed a well-defined separation of layers. Furthermore, the elemental concentration analysis depicts the atomic and weight percentage of individual elements in the structure of MXene. **c** EDS mapping of Ti_3_C_2_T_x_ nanosheets showing distribution of chemical elements in its composition. This analysis confirmed that titanium, fluorine, carbon, and oxygen are the main chemical elements.


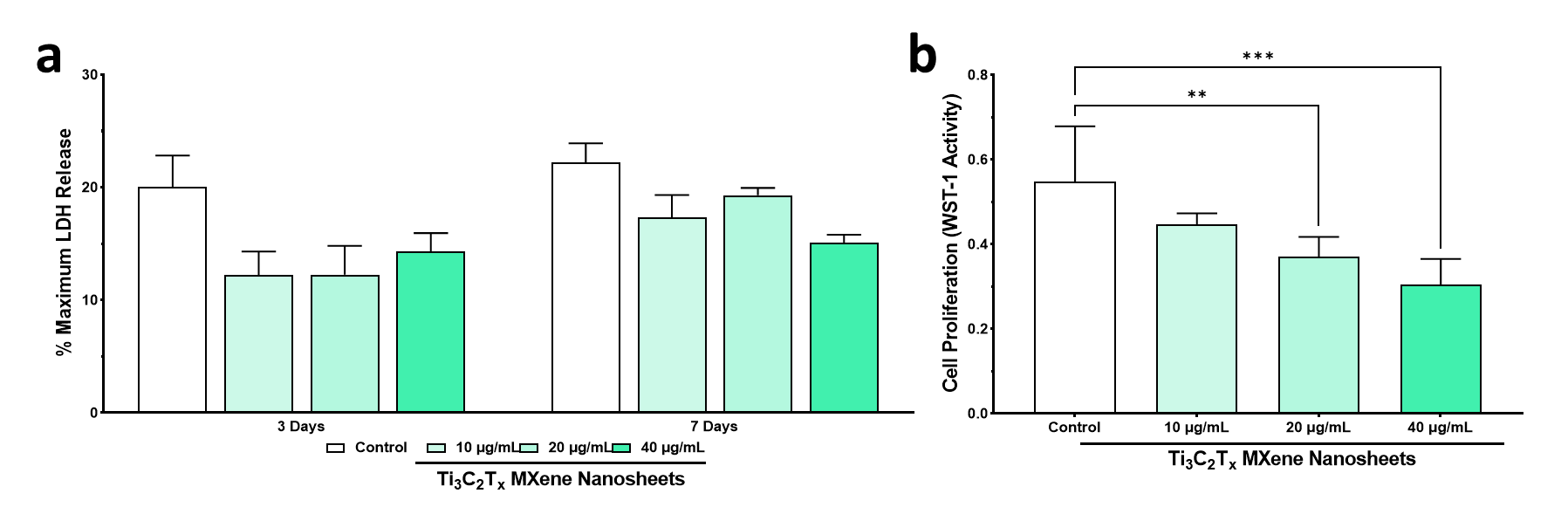


**Supplementary Figure S2. Effect of Ti_3_C_2_T_x_ MXene nanosheets on endothelial cells at higher concentrations. a** At doses up to 40 μg/mL**,** Ti_3_C_2_T_x_ MXene nanosheets did not exert significant cytotoxicity on human umbilical vein endothelial cells (HUVECs). **b** However, HUVECs did display reduced proliferation at doses beyond 20 μg/mL.

**
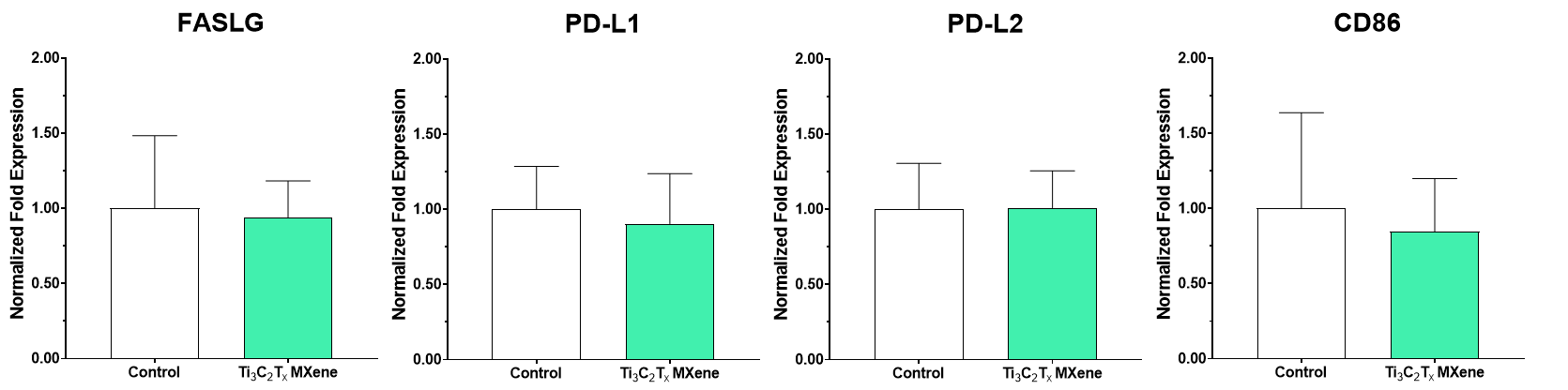
**

**Supplementary Figure S3. Endothelial expression of immune system co-activation or co-inhibition signals.** No significant differences were observed in the expression of the co-activator CD86 and the co-inhibitors FASLG, PD-L1 and PD-L2 between control samples and those treated with 2 µg/mL of Ti_3_C_2_T_x_ MXene nanosheets. Six biological replicates were included per treatment group.


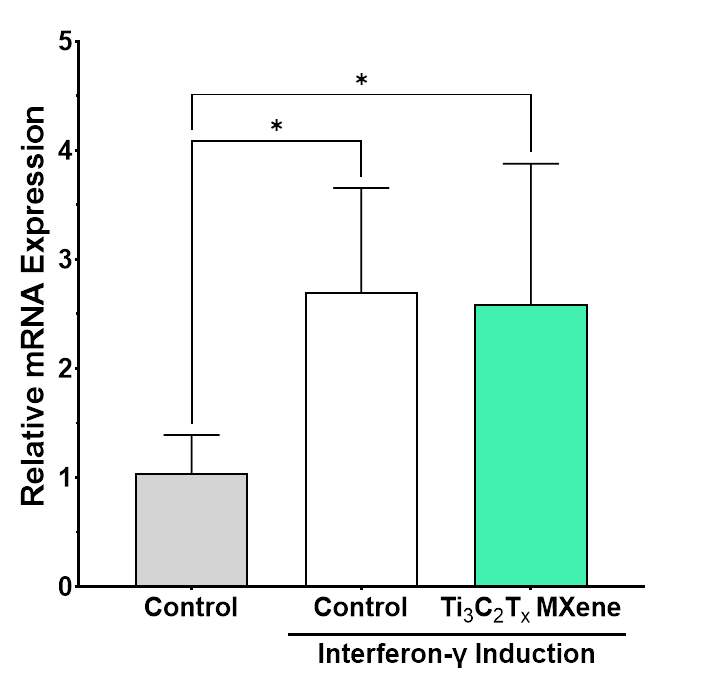


**Supplementary Figure S4. Cytokine-induced upregulation of VCAM-1 in endothelial cells.** HUVECs treated with 2 μg/mL of Ti_3_C_2_T_x_ MXene retained their endothelial phenotype and exhibited significant upregulation of VCAM-1 when exposed to 10 units/mL of IFN-γ. Six biological replicates were included per group.


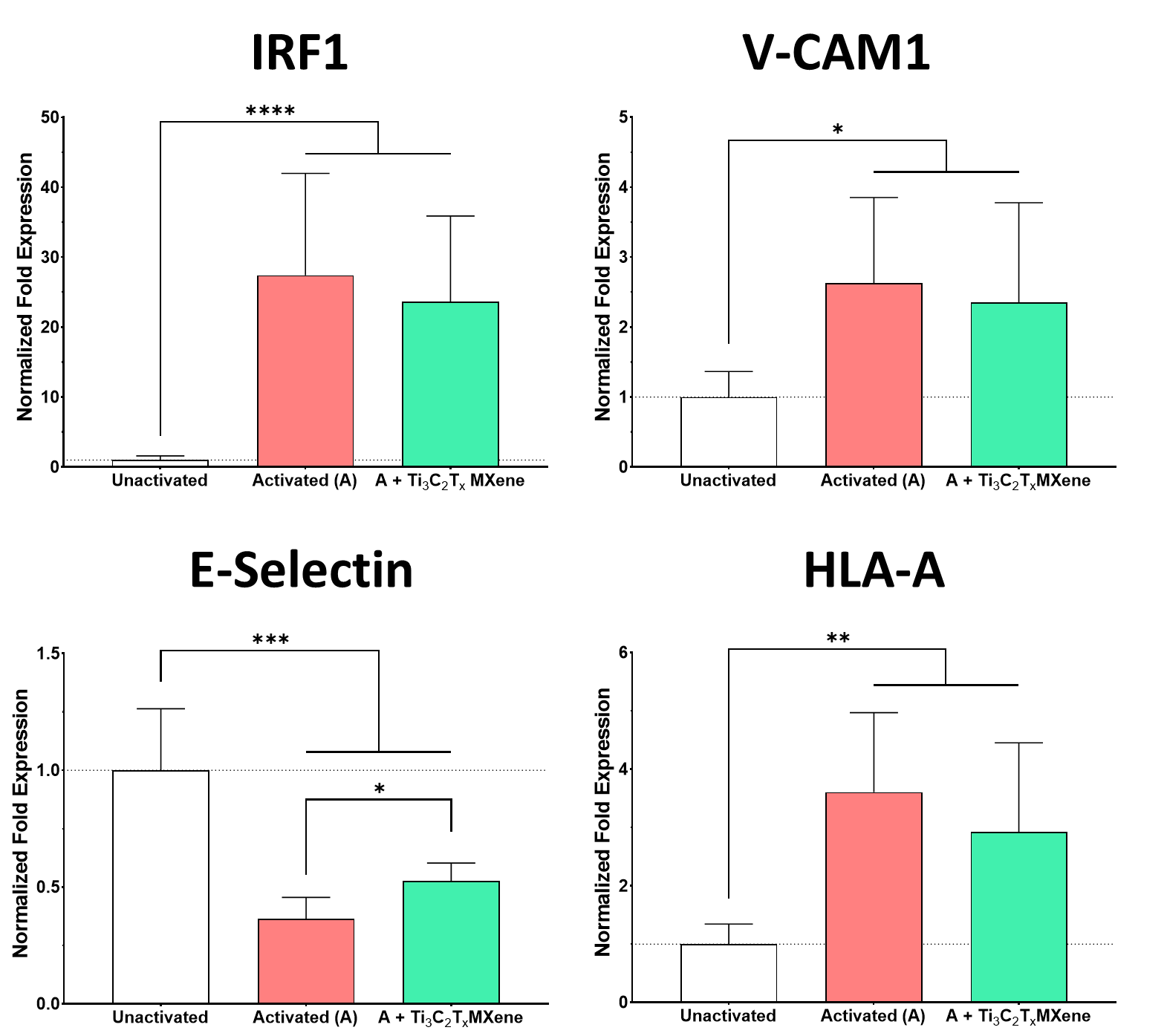


**Supplementary Figure S5. Effects of 2 µg/mL Ti_3_C_2_T_x_ MXene nanosheets on endothelial cells treated with interferon-γ.** Quantitative PCR was performed on HUVECs that were sequentially treated with 10 units/mL IFN-γ and then 2 µg/mL Ti_3_C_2_T_x_ MXene nanosheets. These HUVECs showed significant changes in gene expression after treatment with IFN-γ, including upregulation of the pro-inflammatory genes IRF1, V-CAM1, and HLA-A, and downregulation of E-Selectin. These changes were partially ameliorated after treatment with Ti_3_C_2_T_x_ MXenes, with consistent trends towards decrease in the expression of pro-inflammatory genes and significant increase in the expression of E-Selectin towards baseline levels.


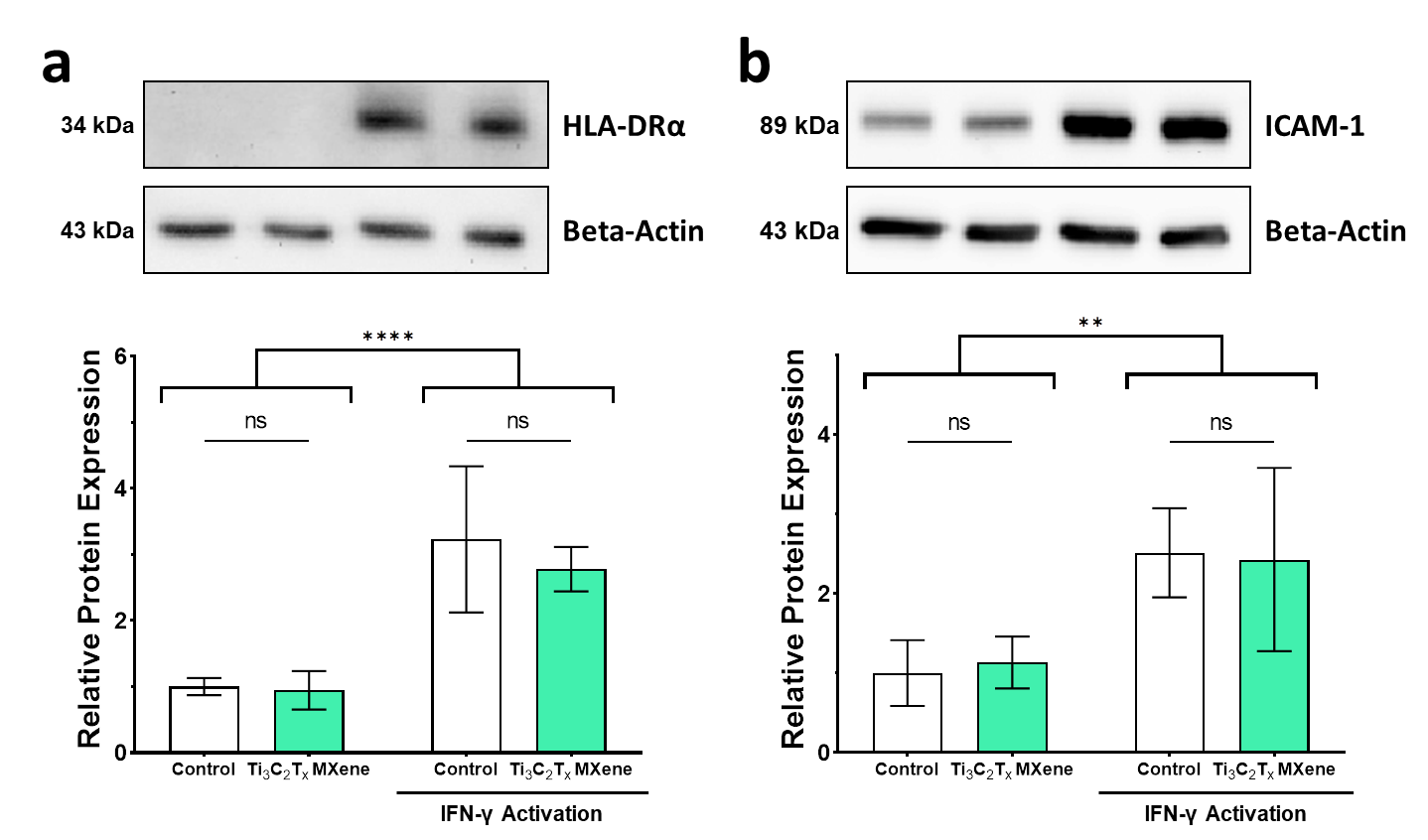


**Supplementary Figure S6. Interferon-γ induced upregulation of endothelial antigen presentation and leukocyte adhesion. a,b** HUVECs treated with 10 units/mL of IFN-γ exhibited robust upregulation of HLA-DRα and ICAM-1. Treatment with 2 μg/mL of Ti_3_C_2_T_x_ MXene did not blunt the HUVECs’ response to interferon-gamma with respect to these two genes. Four biological replicates were included per group.


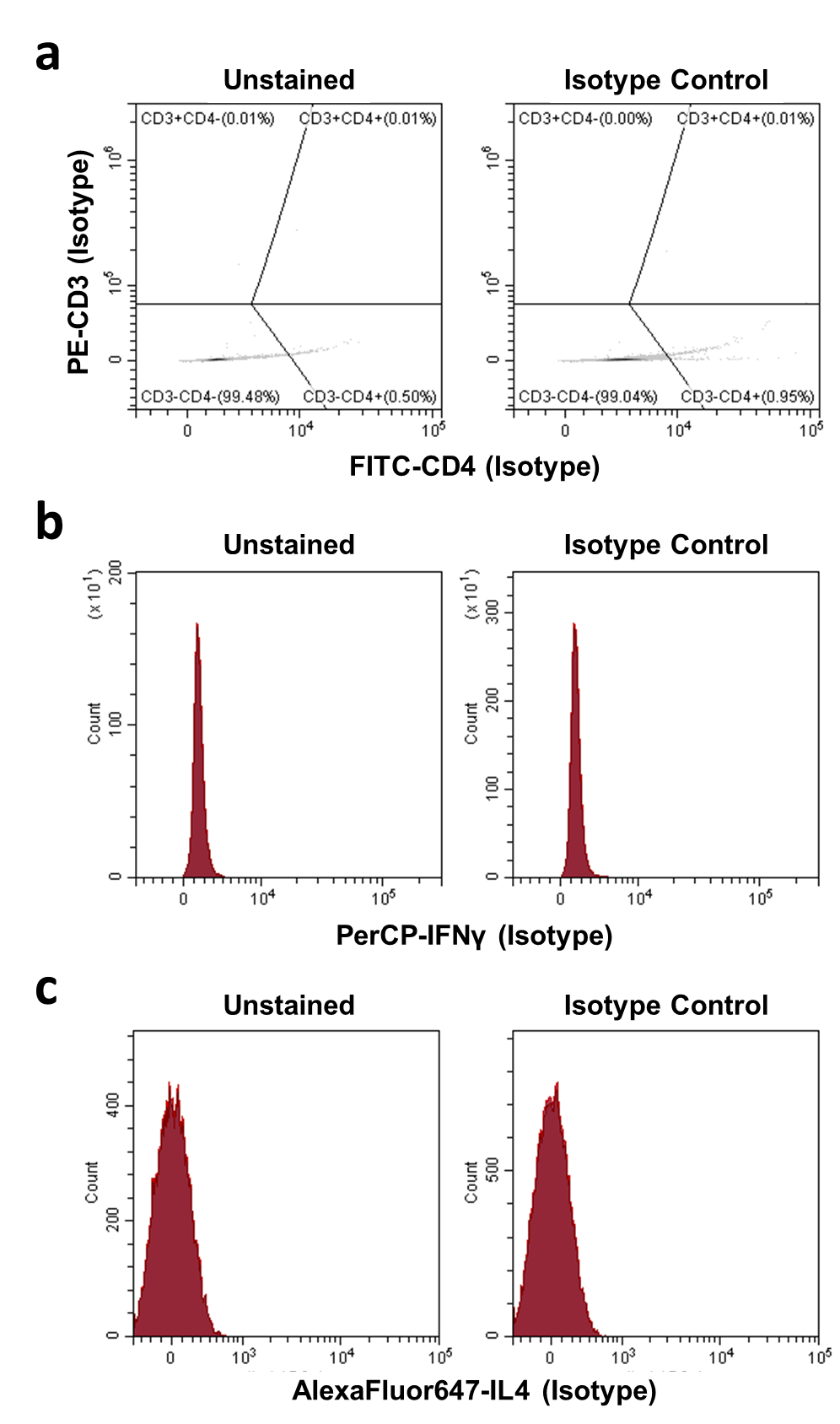


**Supplementary Figure S7. Isotype controls for flow cytometry. a** Isotype controls for FITC-CD4, PE-CD3, **b** PerCP-IFNγ, and **c** AF647-IL4 were used at the same concentrations as their respective antibodies and did not reveal any non-specific binding by lymphocytes.

**
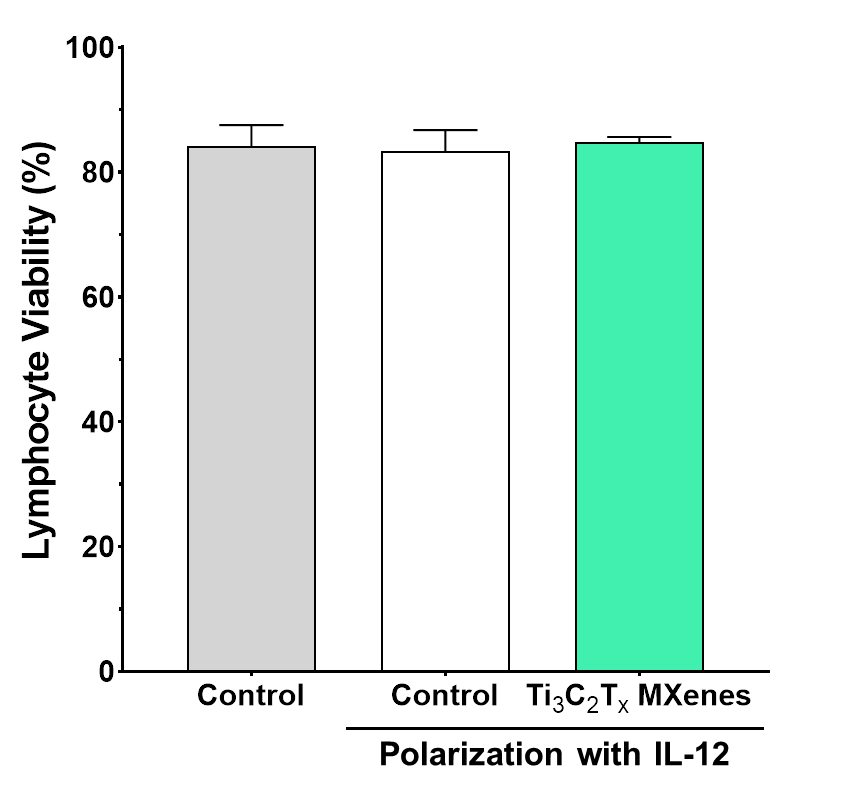
**

**Supplementary Figure S8. Viability of lymphocytes in co-culture with Ti_3_C_2_T_x_ MXene nanosheets.** Treatment with 2 μg/mL of Ti_3_C_2_T_x_ MXene nanosheets did not result in significant changes to the viability of co-cultured lymphocytes. Five biological replicates were included per group.


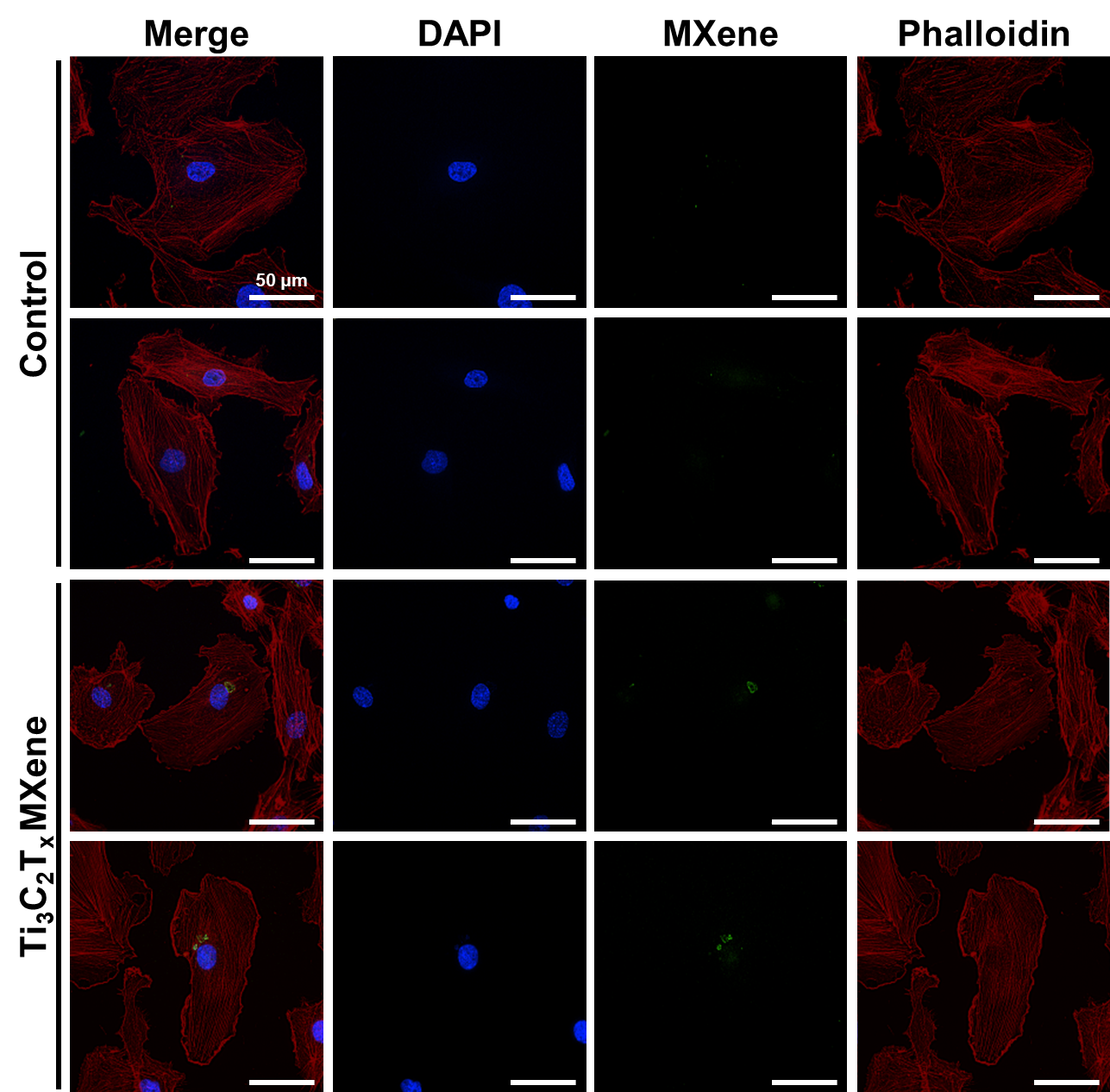


**Supplementary Figure S9. Endothelial uptake of Ti_3_C_2_T_x_ MXene nanosheets.** Ti_3_C_2_T_x_ MXene nanosheets at 2 μg/mL were co-cultured with HUVECs for 72 hours. Cells were then stained with phalloidin (red) and DAPI (blue) and visualized using a fluorescence microscope at 90× magnification. The autofluorescent MXene (green) was uptaken into HUVECs and localized around the nucleus.


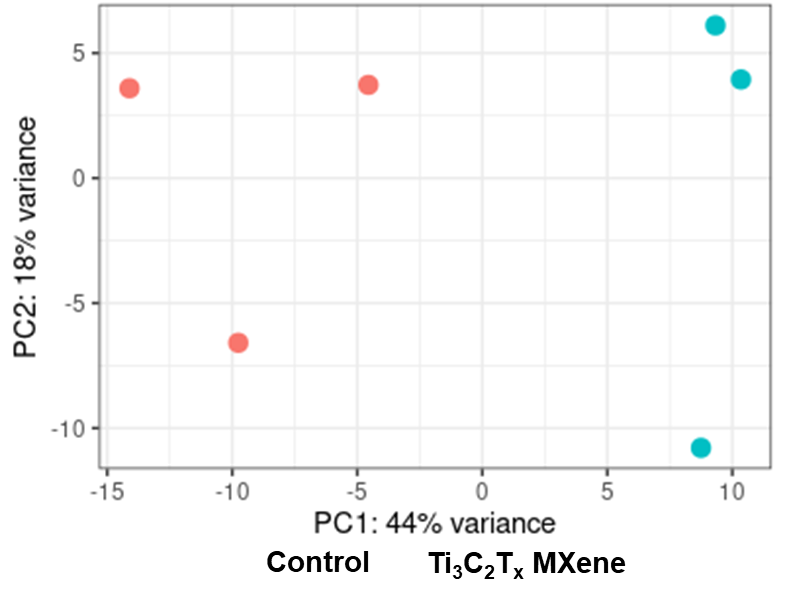


**Supplementary Figure S10. Principal component analysis from RNA sequencing of lymphocytes co-cultured with Ti_3_C_2_T_x_ MXene-treated endothelial cells.** The two treatment groups separated into well-defined clusters along the first principal component, which accounted for 44% of the variance between groups. Three biological replicates were included per group.


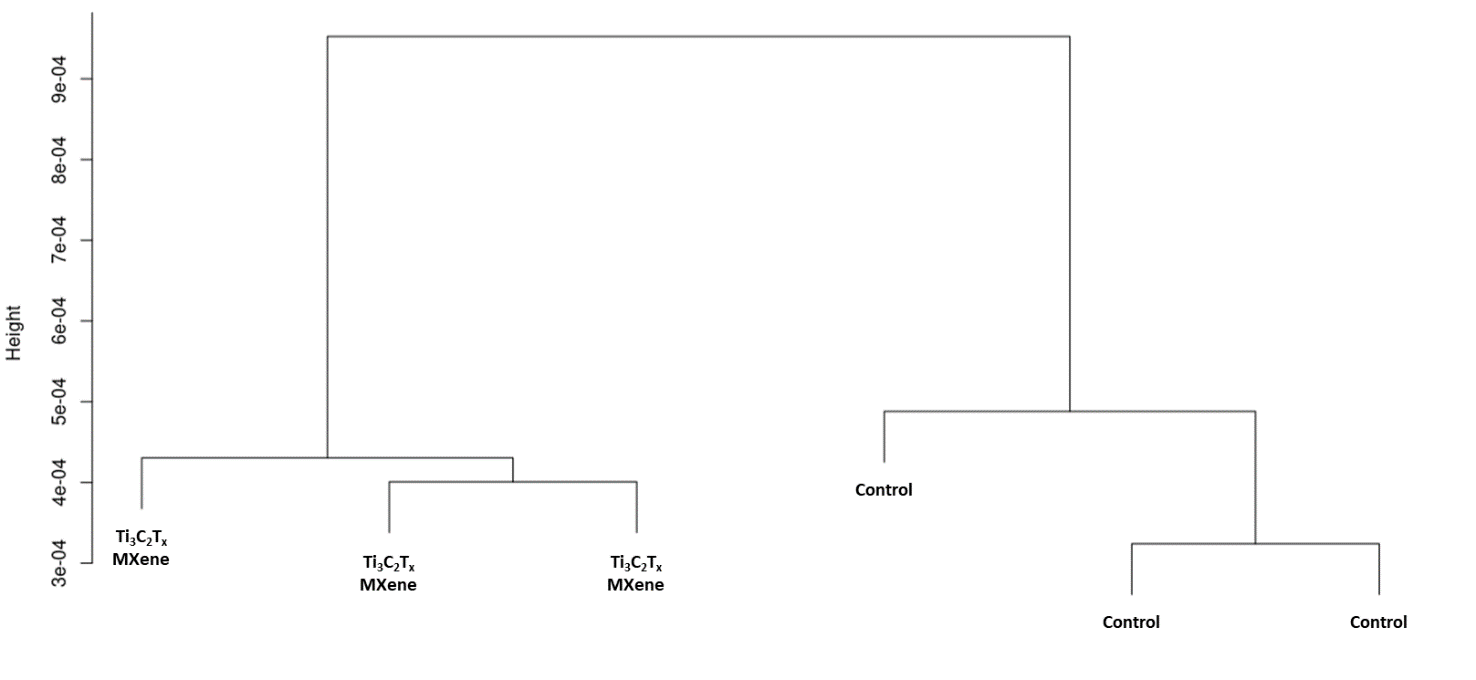


**Supplementary Figure S11. Cluster dendrogram of individual RNA sequencing samples.** Hierarchical clustering was performed using Pearson’s correlation distance and showed compact and well-defined clusters. Three biological replicates were included per group.

**
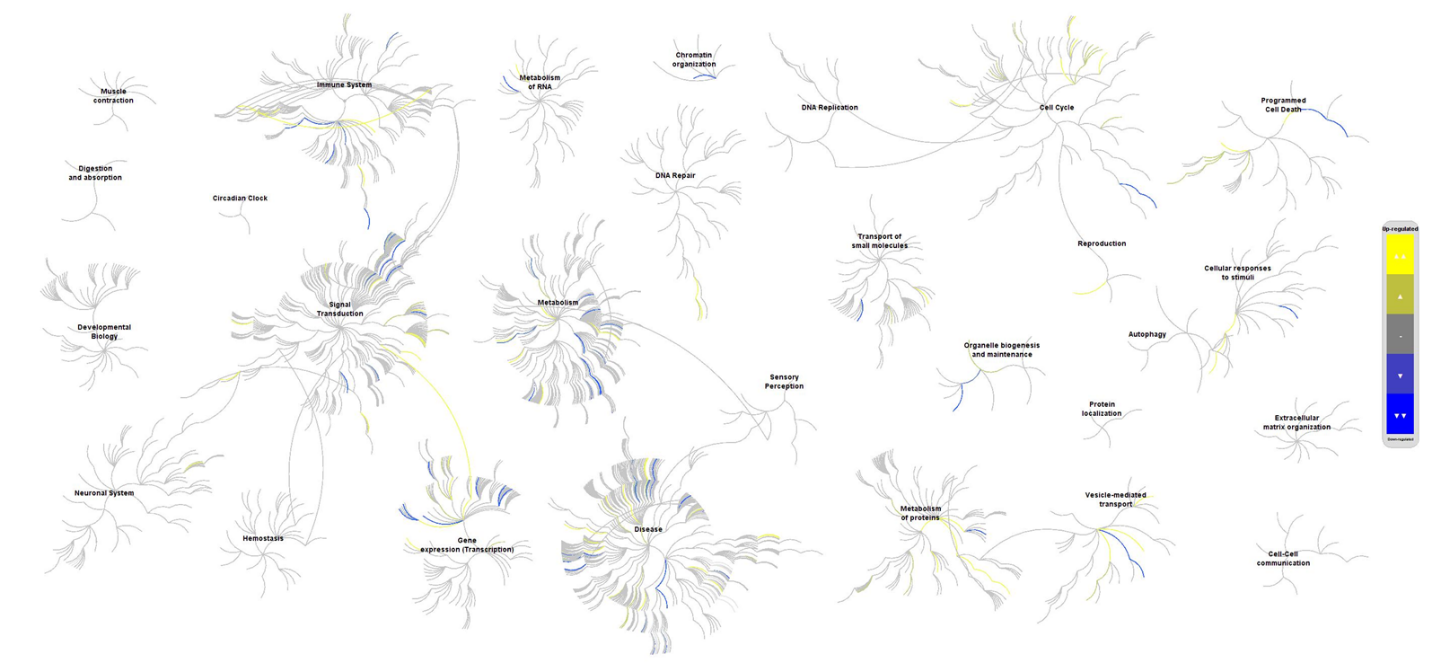
**

**Supplementary Figure S12. Gene set enrichment analysis of RNA sequencing samples.** Gene set enrichment analysis was performed using GSEA and visualized using REACTOME. Amongst these, 1,026 REACTOME gene sets, 392 upregulated gene sets and 6 downregulated gene sets were significantly enriched at FDR <0.05.


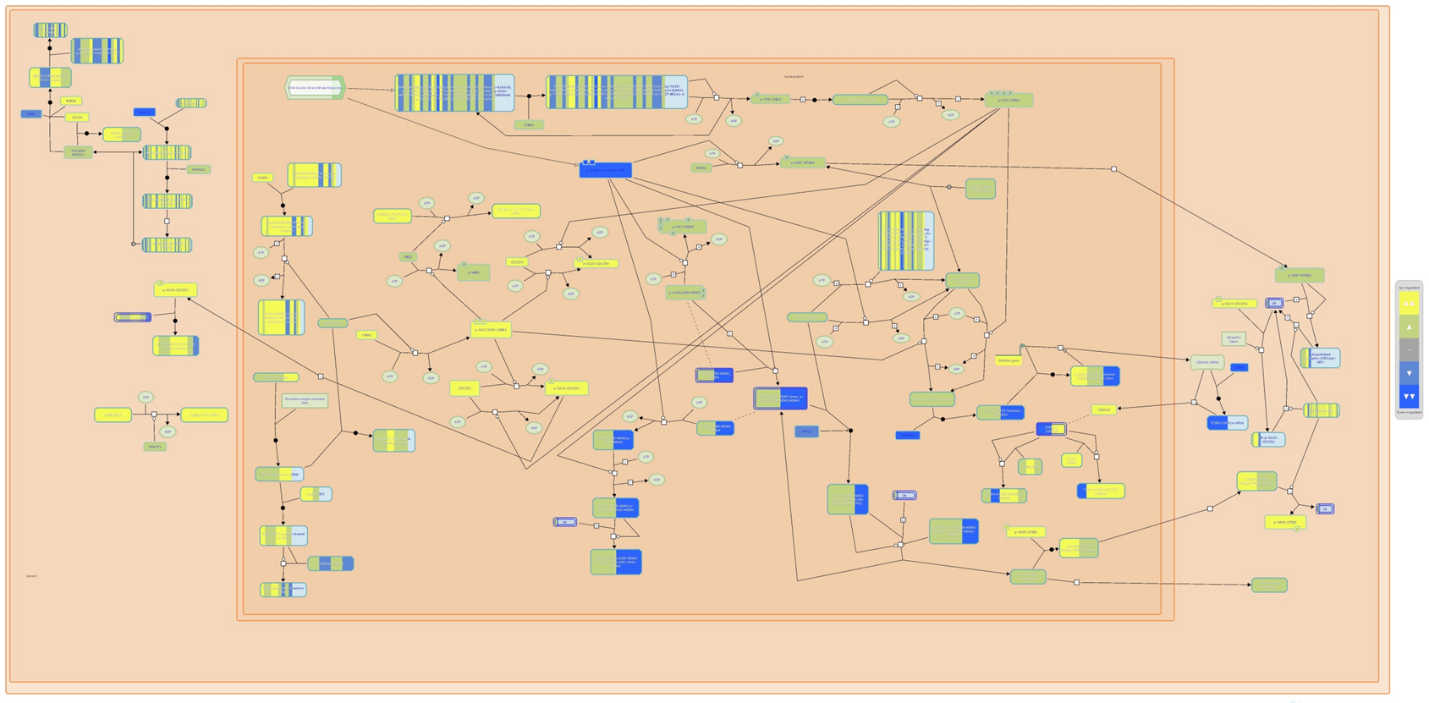


**Supplementary Figure S13. Gene expression changes in cell cycle checkpoints within lymphocytes co-cultured with Ti_3_C_2_T_x_ MXene-treated endothelial cells, based on RNA sequencing data.** REACTOME pathway for cell cycle checkpoints (RIA-69620) showed significant gene expression changes in lymphocytes co-cultured with Ti_3_C_2_T_x_ MXene-treated endothelial cells, compared to those co-cultured with untreated endothelial cells. Three biological replicates were included per group.

**Supplementary Figure S14. Gene expression changes in p21^Cip1^.** Lymphocytes co-cultured with Ti_3_C_2_T_x_ MXene-treated endothelial cells had significantly higher expression of p21^Cip1^, which is implicated in the inhibition of cell cycle progression. Three biological replicates were included per group.


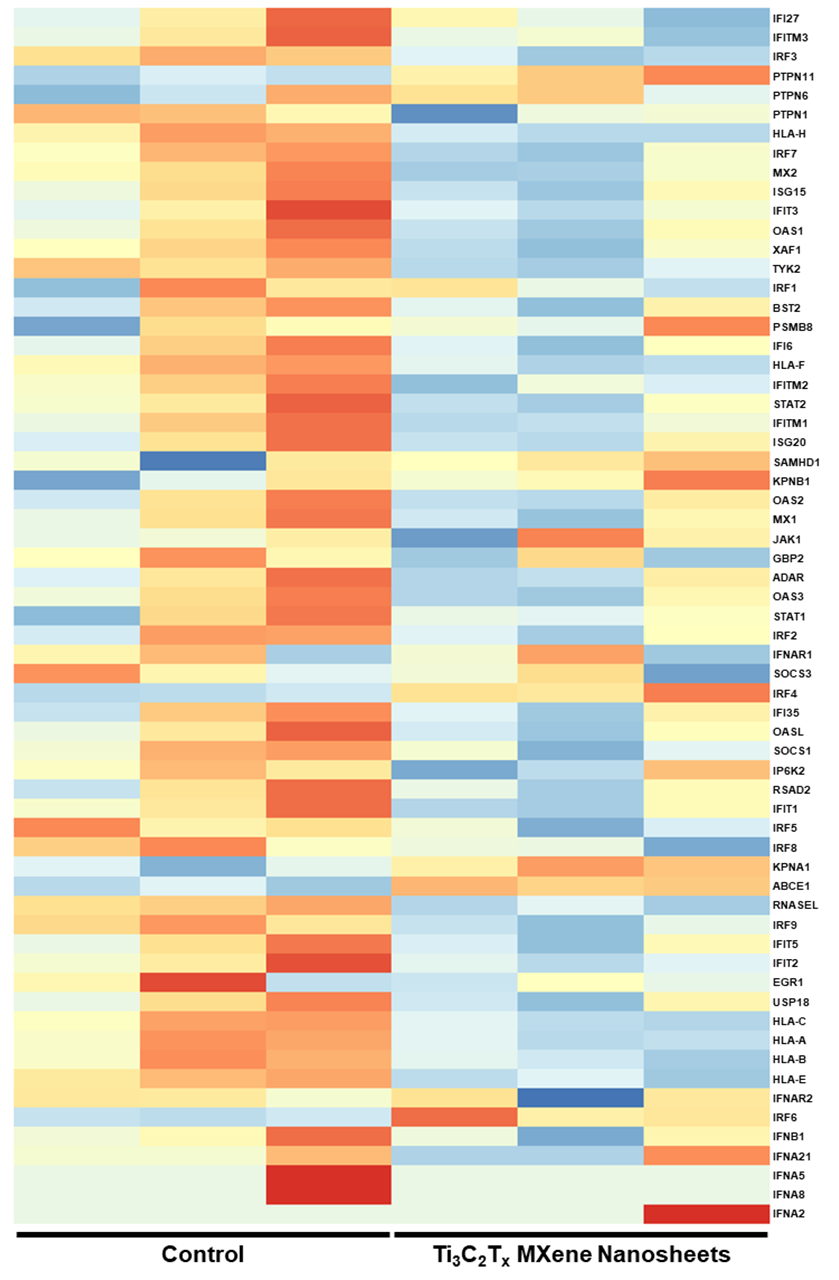


**Supplementary Figure S15. Gene expression changes in interferon alpha/beta signaling within lymphocytes co-cultured with Ti_3_C_2_T_x_ MXene-treated endothelial cells, based on RNA sequencing data.** The heat map shows the relative expression of each gene in the interferon alpha/beta signaling pathway within each replicate sample. Lymphocytes co-cultured with Ti_3_C_2_T_x_ MXene-treated endothelial cells had significantly lower expression of many of these genes. Three biological replicates were included per group.

**
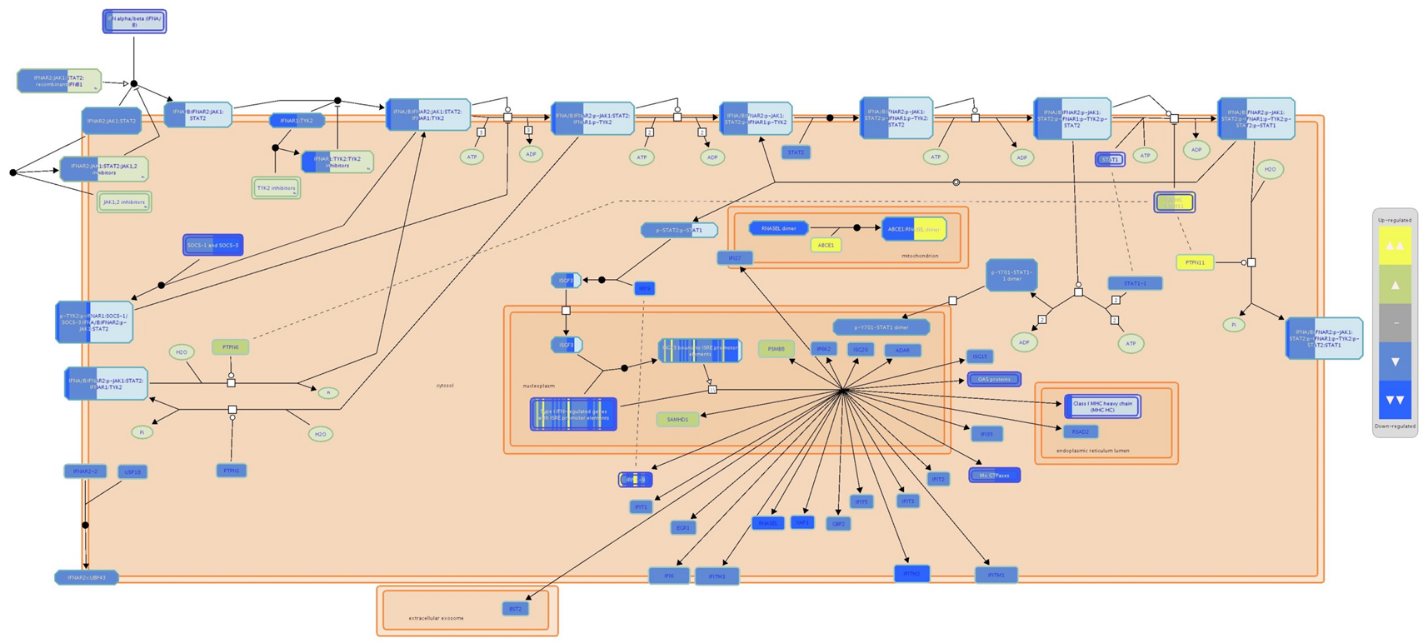
**

**Supplementary Figure S16. REACTOME pathway for interferon alpha/beta signaling.** The REACTOME pathway (I-HSA-909733) showed significant gene expression changes in lymphocytes co-cultured with Ti_3_C_2_T_x_ MXene-treated endothelial cells, compared to those co-cultured with untreated endothelial cells. Three biological replicates were included per group.


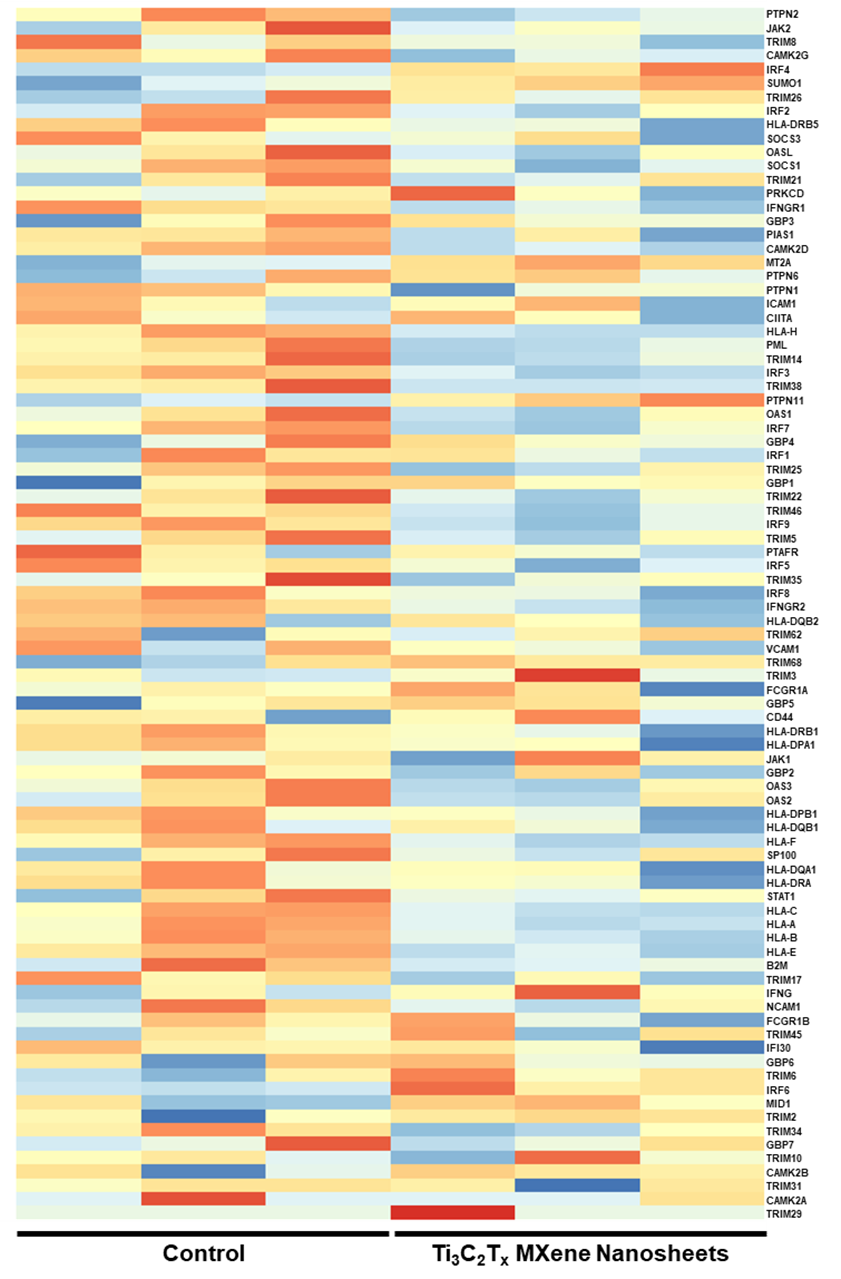


**Supplementary Figure S17. Gene expression changes in interferon gamma signaling within lymphocytes co-cultured with Ti_3_C_2_T_x_ MXene-treated endothelial cells, based on RNA sequencing data.** The heat map shows the relative expression of each gene in the interferon gamma signaling pathway within each replicate sample. Lymphocytes co-cultured with Ti_3_C_2_T_x_ MXene-treated endothelial cells had significantly lower expression of many of these genes. Three biological replicates were included per group.

**
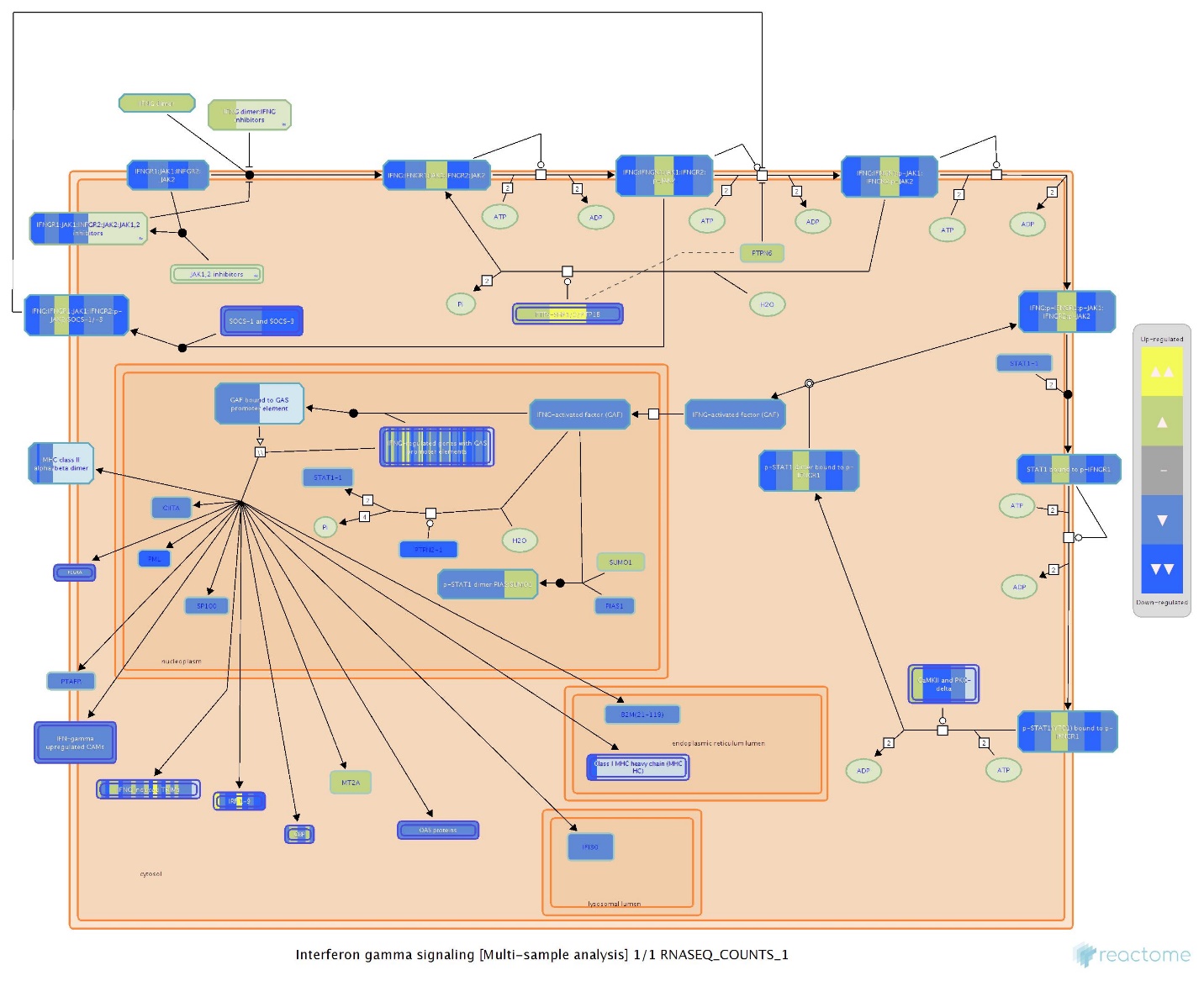
**

**Supplementary Figure S18. REACTOME pathway for interferon gamma signaling.** REACTOME pathway for interferon gamma signaling (R-HSA-877300) showed gene expression changes in lymphocytes co-cultured with Ti_3_C_2_T_x_ MXene-treated endothelial cells, compared to those co-cultured with untreated endothelial cells. Three biological replicates were included per group.


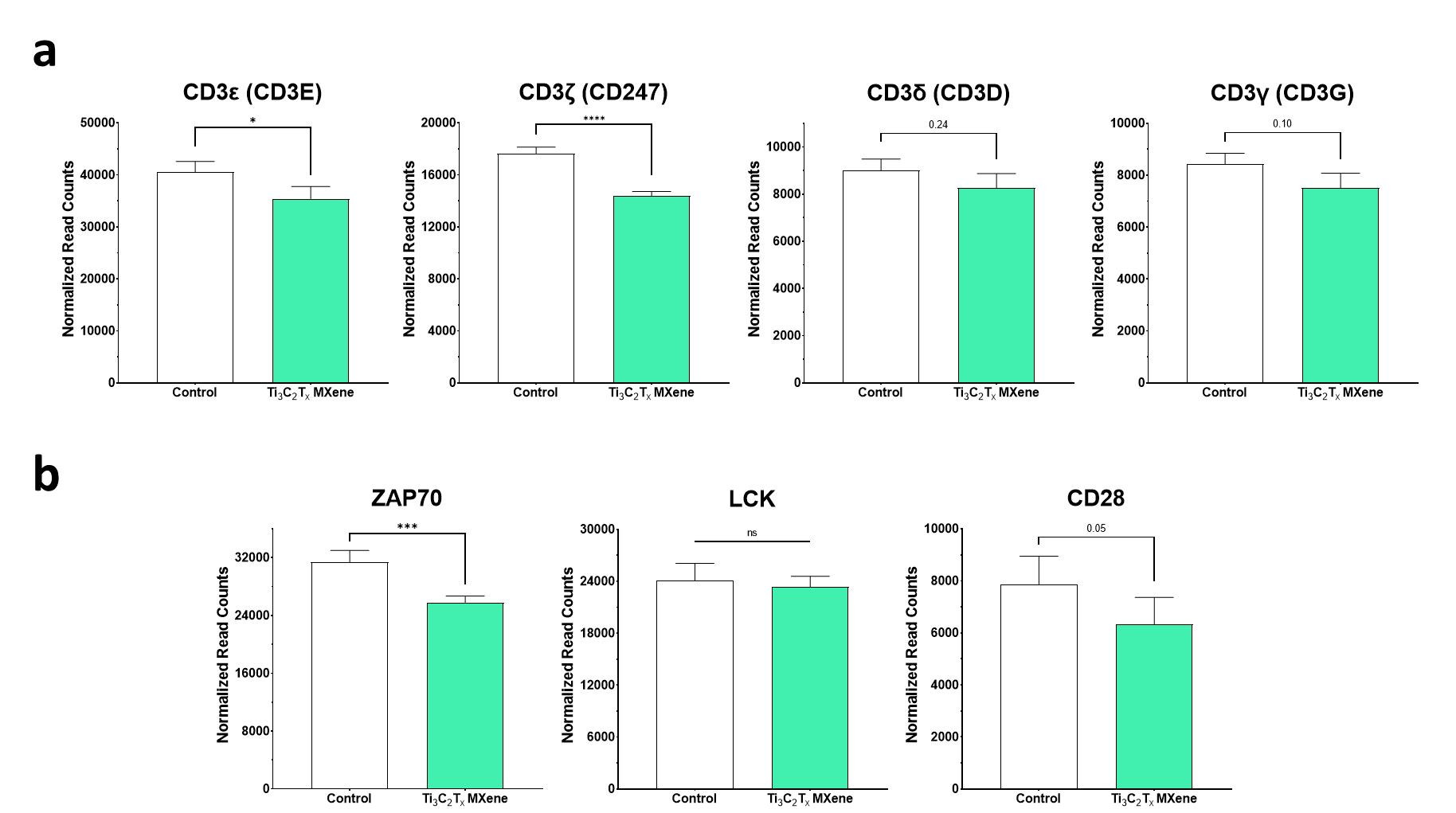


**Supplementary Figure S19. Downregulation of the T-cell receptor, co-stimulator, and its associated tyrosine kinase within lymphocytes co-cultured with Ti_3_C_2_T_x_ MXene-treated endothelial cells, based on RNA sequencing data.** **a** In particular, the invariant T-cell receptor proteins CD3ε (CD3E) and CD3ζ (CD247) were significantly downregulated within lymphocytes co-cultured with Ti_3_C_2_T_x_ MXene-treated endothelial cells, and downregulatory trends were observed in CD3δ (CD3D) and CD3γ (CD3G). **b** Additionally, the key TCR activation-associated tyrosine kinase ZAP-70 was also significantly downregulated within lymphocytes co-cultured with Ti_3_C_2_T_x_ MXene-treated endothelial cells, and a downregulatory trend was observed in the T-cell co-activation receptor CD28. Three biological replicates were included per group.

**
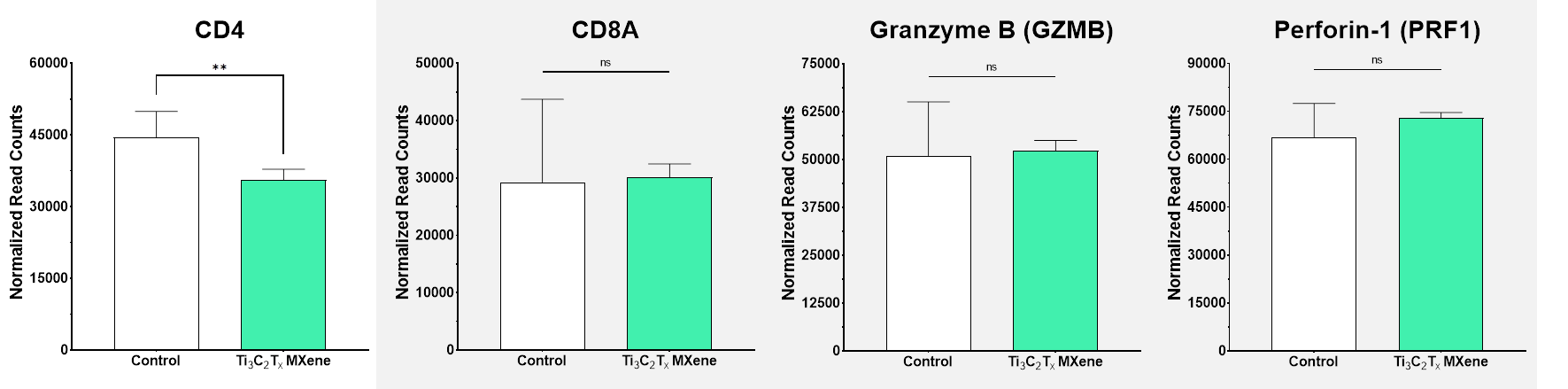
**

**Supplementary Figure S20. Expression of the CD4 and CD8 T-cell co-receptors, as well as key CD8^+^ T-cell associated cytotoxins, based on RNA sequencing data.** Significantly lower expression of the CD4 TCR co-receptor within lymphocytes co-cultured with Ti_3_C_2_T_x_ MXene-treated endothelial cells, while no significant differences were noted in components of cytotoxic CD8^+^ T-lymphocytes. Three biological replicates were included per group.

**
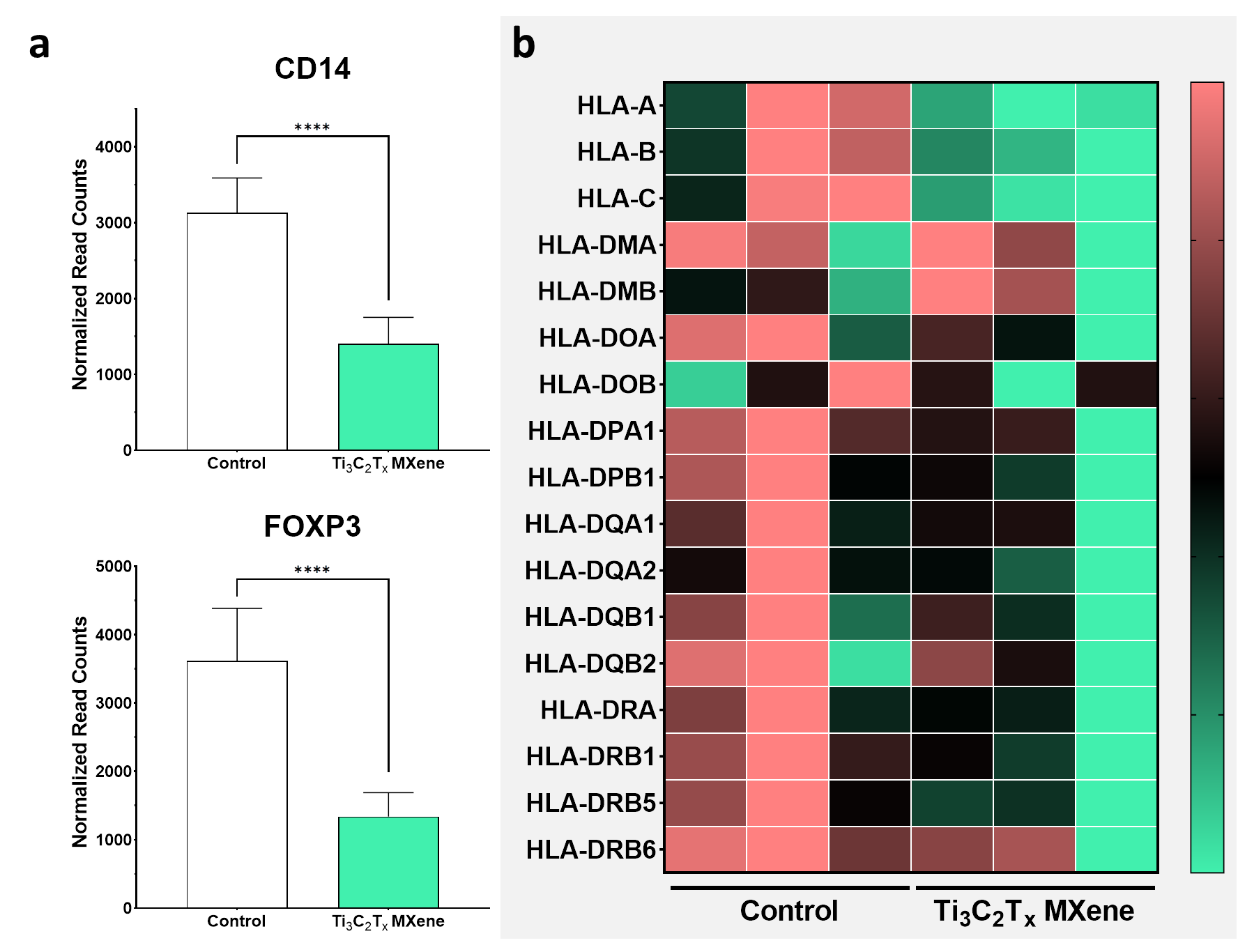
**

**Supplementary Figure S21. Expression of macrophage markers, HLA class I and II, and FOXP3, based on RNA sequencing data.** Significant decreases were observed in the expression levels of the macrophage marker CD14, HLA class I and class II, as well as the regulatory protein FOXP3 in lymphocytes co-cultured with Ti_3_C_2_T_x_ MXene-treated endothelial cells. These changes are consistent with an overall phenotype of reduced acute vascular rejection. Three biological replicates were included per group.


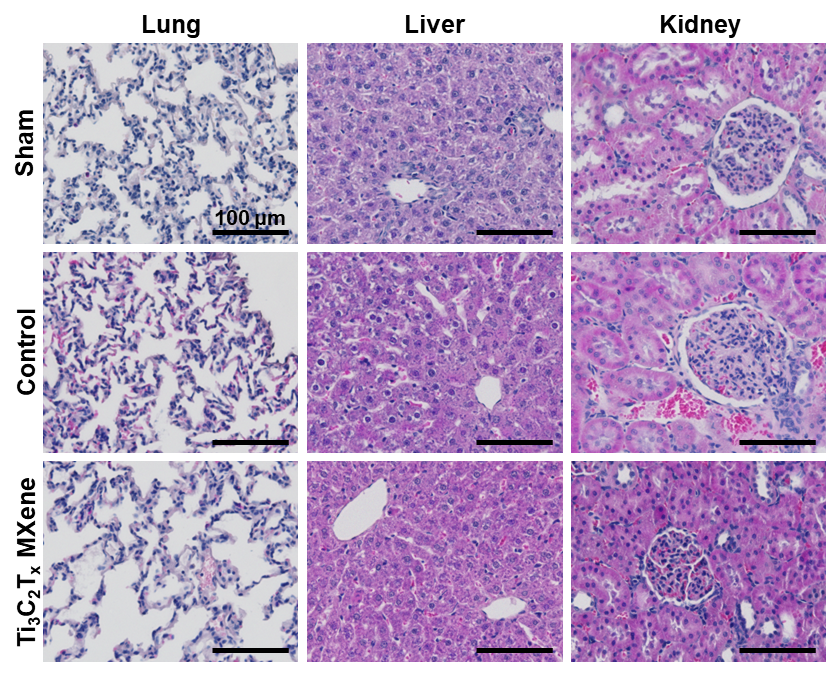


**Supplementary Figure S22. H&E staining of lungs, livers, and kidneys from rats after tail vein injection of Ti_3_C_2_T_x_ MXene nanosheets.** No histologic changes were observed in the lungs, liver, and kidney of animals receiving Ti_3_C_2_T_x_ MXene nanosheets. This further supports the excellent biocompatibility of Ti_3_C_2_T_x_ MXene nanosheets at the doses used for this study.


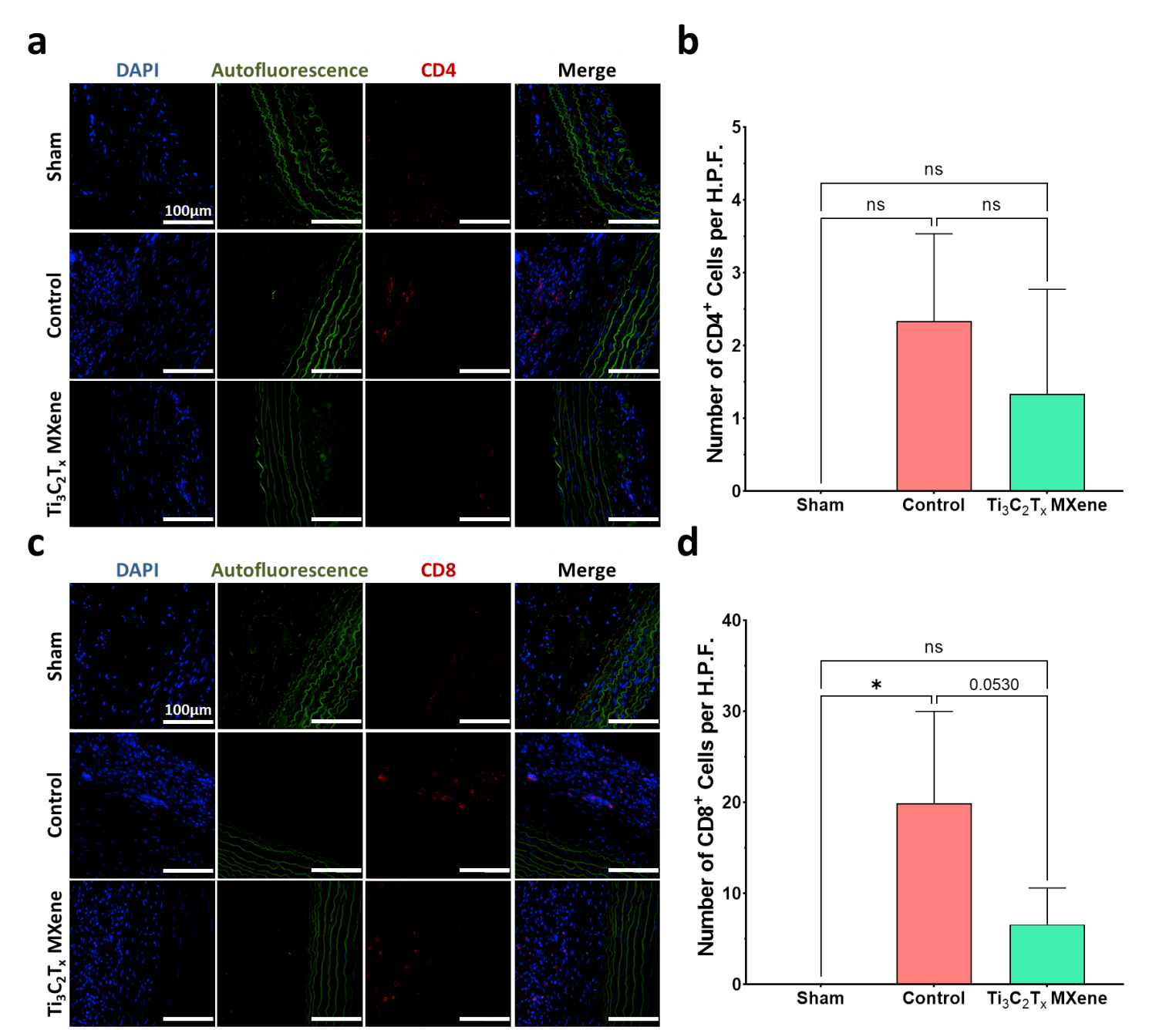


**Supplementary Figure S23. CD4^+^ and CD8^+^ lymphocyte infiltration in the adventitia of transplanted aortic allografts.** A strong trend was observed towards fewer cytotoxic CD8^+^ T-lymphocytes infiltrating into the allograft adventitia amongst Ti_3_C_2_T_x_ MXene treated animals. Three high-powered fields were selected from each of three to four biological replicates per group.

**
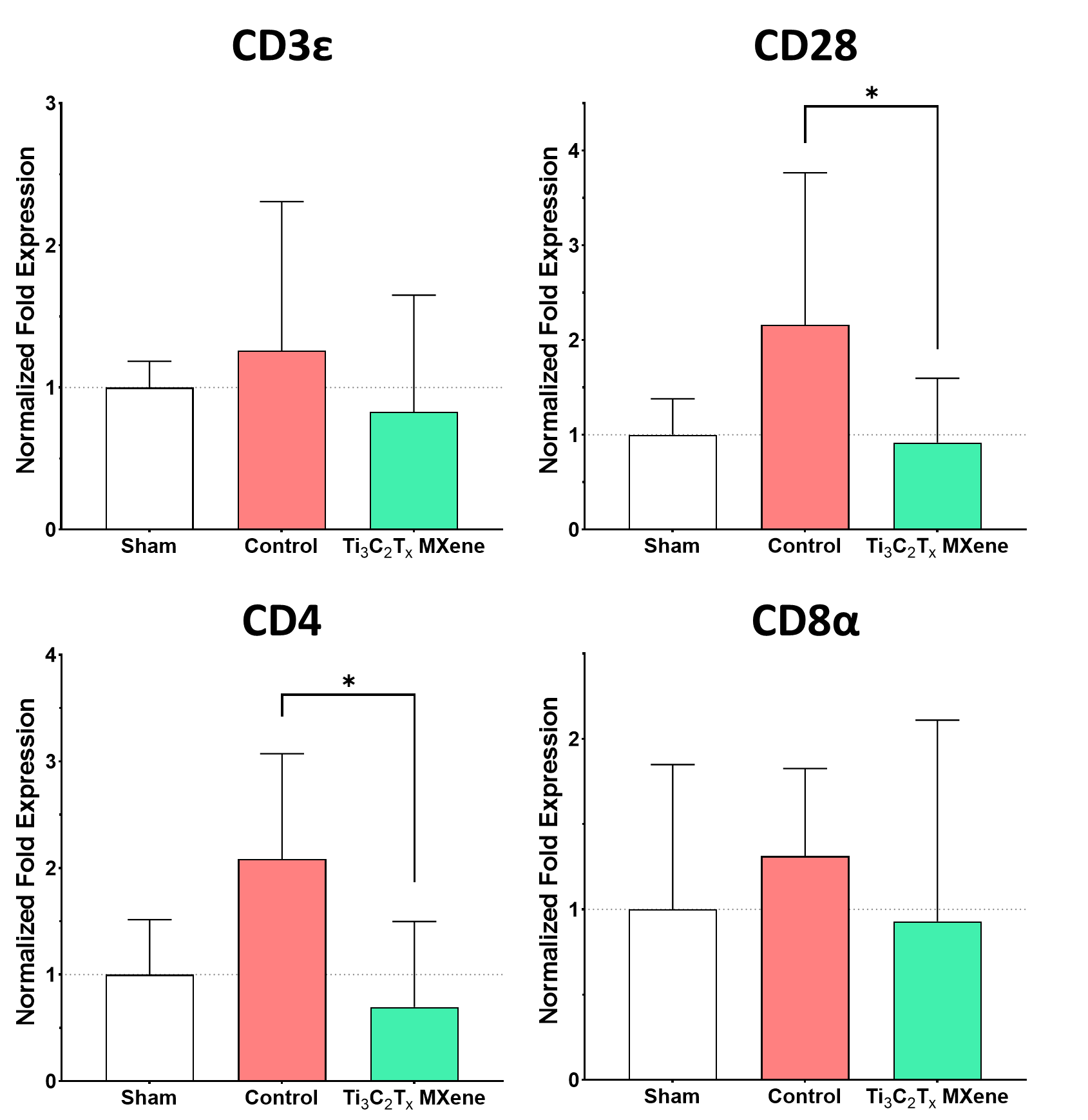
**

**Supplementary Figure S24. Quantitative PCR of peripheral blood mononuclear cells from *in vivo* experiments.** Quantitative PCR of peripheral blood mononuclear cells from the *in vivo* aortic transplantation experiments showed decreases in the expression of major T-cell surface receptors, with significant decreases seen in the expression of CD4 and CD28. This is consistent with *in vitro* experiments which showed decreased T-cell activation and decreased number of CD4^+^ T-lymphocytes.


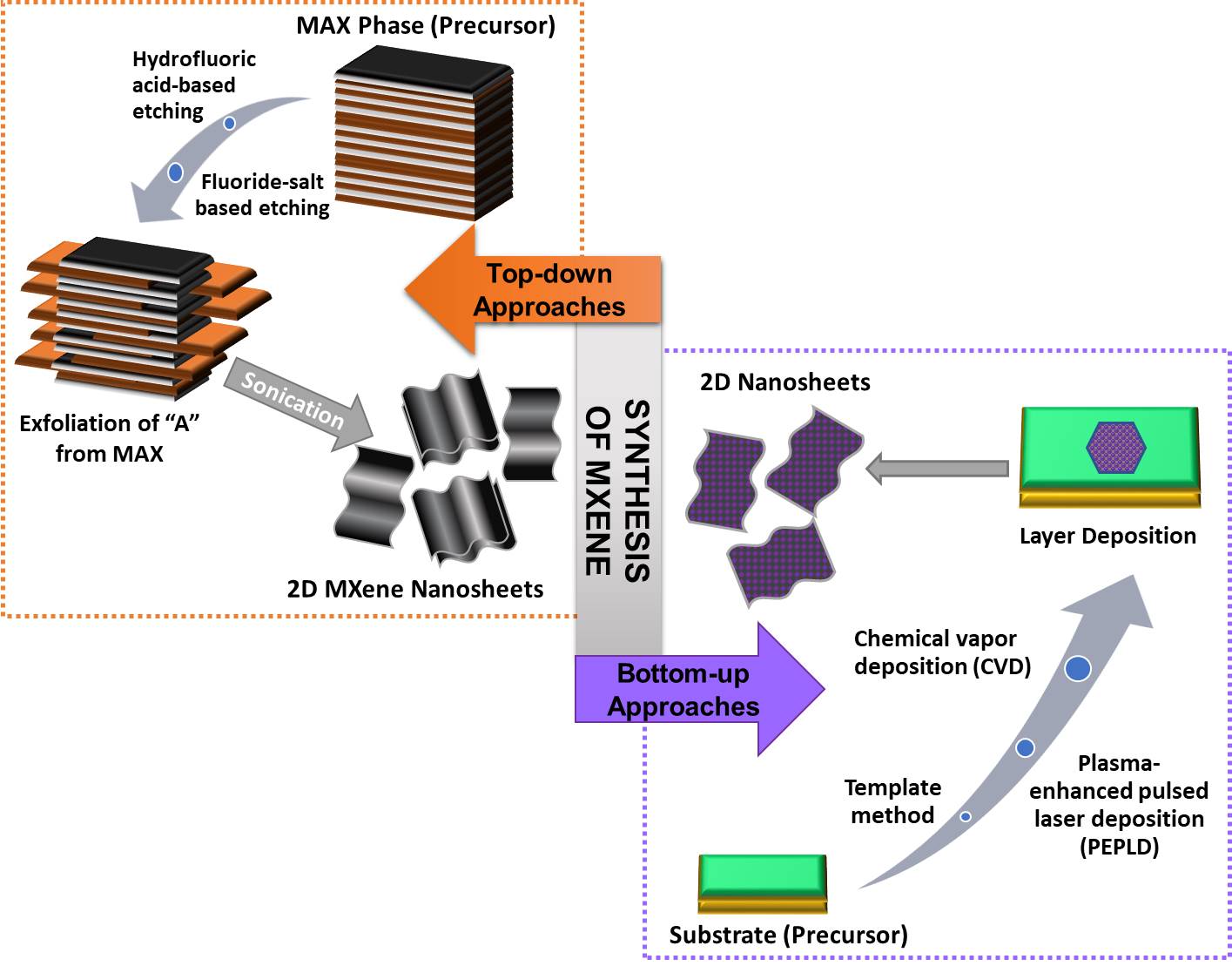


**Supplementary Figure S25. Top-down and bottom-up approaches for synthesis of 2D MXenes.** In top-down methods, bulky MAX phases are selectively etched using different etching agents to form layered MXene nanosheets. This is the preferred method for synthesizing large quantities of MXenes, and the size, surface termination, and defect formation of resultant MXene flakes can be controlled by varying the synthesis parameters. On the other hand, in bottom-up methods, MXene layers are obtained through direct deposition of MXene films on specific substrates. This allows the creation of MXenes with higher crystallinity and stoichiometries that would not be obtainable using a top-down approach.

**Supplementary Table S1.** The XPS peak identification of Ti 2p in Ti_3_C_2_T_x_ MXene nanosheets.

| **Peak** | **Position** | **FWHM** | **Raw Area** | **%At Conc.** |
| --- | --- | --- | --- | --- |
| Ti 2p | 457.8 | 2.41 | 1363.14 | 100 |
| Ti‒C | 457.22 | 0.84 | 185.091 | 13.33 |
| Ti(II) | 457.92 | 1.3 | 454.065 | 32.7 |
| Ti(III)/Ti‒F | 458.99 | 2.08 | 531.078 | 38.24 |
| Ti‒C | 462.33 | 0.84 | 45.7781 | 3.3 |
| Ti(II) | 463.08 | 1.16 | 119.417 | 8.59 |
| Ti(III)/Ti‒F | 463.95 | 1.05 | 53.234 | 3.83 |

**Supplementary Table S2.** The XPS peak identification of C 1s in Ti_3_C_2_T_x_ MXene nanosheets.

| **Peak** | **Position** | **FWHM** | **Raw Area** | **%At Conc.** |
| --- | --- | --- | --- | --- |
| C 1s | 283.8 | 2.32 | 3428.58 | 100 |
| C‒Ti | 281.85 | 0.81 | 214.786 | 6.23 |
| C‒Ti | 282.66 | 1.17 | 765.683 | 22.2 |
| C‒C/CH_2_/CH_3_ | 283.79 | 1.47 | 1805.4 | 52.35 |
| C‒O | 284.84 | 2.48 | 595.792 | 17.27 |
| COOH | 288.82 | 1.05 | 67.0826 | 1.94 |

**Supplementary Table S3.** The XPS peak identification of O 1s in Ti_3_C_2_T_x_ MXene nanosheets.

| **Peak** | **Position** | **FWHM** | **Raw Area** | **%At Conc.** |
| --- | --- | --- | --- | --- |
| O 1s | 531.2 | 2.94 | 15812.3 | 100 |
| TiO_2_ | 527.43 | 0.87 | 268.223 | 1.68 |
| Ti‒O | 528.55 | 1.38 | 1159.4 | 7.27 |
| Ti‒O | 529.87 | 1.56 | 2493.77 | 15.64 |
| C‒O | 531.27 | 1.98 | 8781.92 | 55.06 |
| C=O/H_2_O | 532.4 | 1.96 | 3246.38 | 20.35 |

**Supplementary Table S4.** The XPS peak identification of F 1s in Ti_3_C_2_T_x_ MXene nanosheets.

| **Peak** | **Position** | **FWHM** | **Raw Area** | **%At Conc.** |
| --- | --- | --- | --- | --- |
| F 1s | 684.8 | 4.31 | 8526.82 | 100 |
| Ti‒F | 682.88 | 1.84 | 2729.71 | 31.86 |
| Ti‒F | 684.88 | 2.4 | 5001.8 | 58.37 |
| Al‒F | 686.35 | 1.58 | 837.803 | 9.77 |

**Supplementary Table S5.** Biocompatibility of various MXene compositions.

| **MXene Composition** | **Concentration & Dose Range** | ***In vitro* & *In vivo* Model** | **Bio-Application** | **Toxicity Inference** | **Ref.** |
| --- | --- | --- | --- | --- | --- |
| Ti_3_C_2_T_x_ nanosheets | (100 & 500) µg/mL for 7 days | Human umbilical vein endothelial cells (HUVECs) | Metabolomics approach to evaluate the cytotoxicity of cell-nanomaterial interaction | No significant change in the cell viability was observed between control and Ti_3_C_2_T_x_ treated cells. | [1] |
| Ti_3_C_2_ QDs,  Nb_2_C QDs | (0-100) µg/mL for 24 hours | HUVECs | Cytotoxic evaluation of Ti and Nb based MXenes in cell-nanomaterial interaction | Ti_3_C_2_ QDs induced cytotoxicity at 100 µg/mL whereas Nb_2_C QDs did not show any significant cytotoxicity | [2] |
| Ti_3_C_2_T_x_ nanosheets | (0-100) µg/mL for 7 days | Human mesenchymal  stem cells (hMSCs) | Bone tissue engineering | Ti_3_C_2_T_x_ with concentration >50 µg/mL were cytotoxic to the cells but with concentration <50 µg/mL, it did not show any significant cytotoxicity.  Also, a concentration <20 μg/mL induced & accelerated osteogenic differentiation | [3] |
| Ti_3_C_2_T_x_ nanosheets | (0-25) µg/mL for 24 hours | Neural stem cells  (NSCs)  and NSCs-derived  differentiated cells | Neural tissue engineering | Ti_3_C_2_T_x_ with concentration >25 µg/mL were cytotoxic but was nontoxic at a concentration of 12.5 µg/mL | [4] |
| (Single, few, and multilayered Ti_3_C_2_T_x_ nanosheets), TiC, Ti_2_AlC, and Ti_3_AlC_2_ MAX phases | (10-400) µg/mL for 24 & 48 hours | Human fibroblast cell line MSU1.1 | Bioinstrumentation | Concentration dependent cytotoxicity was experienced in MAX phases.  Layered MXenes showed no cytotoxicity compared to the MAX phases | [5] |
| Nb_2_CT_x_ nanosheets | (0-200) µg/mL for 24 hours | Murine breast cancer 4T1 cell line and U87 cell line | Photothermal therapy | Non-toxic towards cells even at 200 µg/mL with laser intervention | [6] |
| Ti_2_N-MXene QDs | (0-80) ppm for 24 hours | 4T1, U87, and 293T cells | Photothermal therapy | Ti_2_N QDs possess excellent biocompatibility | [7] |
| Ti_3_C_2_T_x_ | (25-200) µg/mL for 96 hours post-fertilization | Zebrafish embryo model | Biomedical applications | Ti_3_C_2_T_x_ with concentration (50 and 100) µg/mL showed no signs of teratogenic and neurotoxic effects, and no harmful effects in neuromuscular activities | [8] |
| Ti_3_C_2_T_x_ | (0-200) µg/mL for 4 hours | Vero E6 cells | Anti-viral and Immunomodulatory agents | Showed no-toxicity towards cells up to concentration of 200 µg/mL | [9] |
| Ta_4_C_3_T_x_ MQDs | (2-100) µg/mL for 7 days | HUVECs | Immunomodulatory agents for vascular allopathy treatment | No significant differences were observed in cellular proliferation between the MQD-treated groups and the control | [10] |
| Ti_3_C_2_ MQDs | 66ng/mL to 1.32 µg/mL for 24 hours | Human iPSC-derived fibroblasts & rat bone-marrow derived mesenchymal stem cells | Immunomodulatory agents | No significant cytotoxicity was observed between the control and MQDs treated group | [11] |
| Ti_3_C_2_ MQDs | (10-20) µg/mL for 24 hours | RAW264.7 | Bioimaging | No significant cytotoxicity was observed between the control and MQDs treated group | [12] |
| Nitrogen and phosphorus functionalized Ti_3_C_2_ MQDs | (0-100) µg/mL for 24 hours | THP-1 monocytes | Bioimaging | Showed modest cytotoxicity at lower concentrations ranging below 25µg/mL | [13] |
| V_2_C MXenzyme | (0-400) µg/mL for 24 hours | L929 and PC12 cells | Ros scavengers for neurodegenerative treatments | Showed no visible cytotoxicity to cells even when treated with 200µg/mL concentration | [14] |

[1] Carbon, 178, 810-821.

[2] Journal of Applied Toxicology, 41(5), 745-754.

[3] Materials, 14(16), 4453.

[4] Chemical Research in Toxicology, 33(12), 2953-2962.

[5] ACS Biomaterials Science & Engineering, 5(12), 6557-6569.

[6] Journal of the American Chemical Society, 139(45), 16235-16247.

[7] Chemical Engineering Journal, 400, 126009.

[8] Environmental Science: Nano, 5(4), 1002-1011.

[9] Nano today, 38, 101136.

[10] Advanced Functional Materials, 31(46), 2106786.

[11] Advanced healthcare materials, 8(16), 1900569.

[12] Advanced Materials, 29(15), 1604847.

[13] Nanoscale, 11(30), 14123-14133.

[14] Nature communications, 12(1), 1-16.

**Supplementary Table S6.** Applications of different types of MXene based nanosystems for immunologic applications.

| **Different Type of MXenes** | **Therapeutic Applications** | ***In vitro/vivo* Models** | **Reference** |
| --- | --- | --- | --- |
| 2D Ti_3_C_2_  @  Ti_3_C_2_/metformin/compound polysaccharide system | Immunotherapy/chemotherapy /photodynamic therapy (PDT)/photothermal  therapy (PTT) | MDA-MB-231 tumor-bearing BALB/c nude mice | [1] |
| 2D Nb_2_C  @  Imiquimod R837/Nb_2_C/3D-printing scaffold system | Immunotherapy/PTT | 4T1 tumor-bearing BALB/c mice | [2] |
| 2D Ti_3_C_2_  @  Ti_3_C_2_/gold nanoparticles (AuNPs) system | Immune biosensing | *In vitro* (in human serum for early cancer diagnosis and monitoring) | [3] |
| 2D Ti_3_C_2_  @  Ti_3_C_2_-based interdigitated  capacitance transducer | Immune biosensing (Electrochemical immunoassay of prostate specific antigen) | *In vitro* (in real human serum to detect prostate-specific antigen for protein diagnostics and biosecurity | [4] |
| 2D Ti_3_C_2_  @  Ti_3_C_2_-based composites  With engineered surface | Combined PTT/PDT/  chemotherapy tri-therapy  & Targeted drug delivery approaches for cancer therapy | *In vitro and in vivo* models | [5] |
| 2D Ti_3_C_2_  @  MXene/AuNPs nanocomposites | Enhanced  Photo-Radio Combined Therapy | *In vitro* and *in vivo* rat model of tumor | [6] |
| 2D and 0D Ti_3_C_2_,Ta_4_C_3_, and Nb_2_C  @  MXene and drug/polymer based composites | PTT  with high photothermal conversion properties and magnetic resonance (MR) imaging | *In vitro* and in vivo photothermal ablation  of tumor | [7] |
| 2D Ti_3_C_2_  @  Ti_3_C_2_/doxorubicin | Tumor targeting  photothermal/photodynamic/  chemo  synergistic therapy | Effective cancer cell  killing and tumor tissue destruction *in vitro* &*vivo* | [8] |
| 2D Nb_2_C  @  Nb2C MXene-based composite | Photothermal tumor eradication in NIR‑I and NIR-II biowindows/ Imaging | *In vitro* and *in vivo* tumor model | [9] |
| 2D Ti_3_C_2_  @  Ti_3_C_2_/doxorubicin | PTT/chemotherapy  Tumor therapy | *In vitro* and *in vivo* | [10] |
| 2D Ta_4_C_3_  @  Ta_4_C_3_/MnO_x_-based composite | PTT/ Magnetic resonance imaging/ computerized tomography (CT) for tumor therapy | *In vitro* and *in vivo* tumor model | [11] |
| 0D Ti_3_C_2_  @  Ti_3_C_2_ quantum dots | PTT  Tumor therapy | *In vitro* and *in vivo* | [12] |
| 2D Ti_2_C  @  Ti_2_C-based PEG composites | PTT | *In vitro* tumor model | [13] |
| 2D Ti_3_C_2_  @  Ti_3_C_2_/cobalt nanowire heterojunction | Controlled drug delivery and chemo-photothermal therapy | *In vitro* model | [14] |
| 2D Ta_4_C_3_  @  Ta_4_C_3_/MnO_x_-based composite | Multiple imaging-guided photothermal tumor ablation | *In vitro* and *in vivo*  model | [15] |
| 2D V_2_C  @  V_2_C delaminated nanosheets | Enhanced near-infrared photothermal  for photoacoustic (PA) and MRI- guided PTT of cancer | *In vitro* and *in vivo* tumor model | [16] |
| 0D V_2_C  @  V_2_C-based quantum dots | Delivery for nucleus-target low-temperature photothermal tumor therapy | *In vitro* and *in vivo* model | [17] |
| 2D Mo_2_C  @  Ultrathin Mo_2_/polyvinyl alcohol-based composites | Efficient theory-oriented photonic tumor hyperthermia  applications | *In vitro* and *in vivo* tumor model | [18] |

[1] J. Mater. Chem. B, 2020,8, 6402.

[2] Adv. Funct. Mater.2021, 31, 2006214.

[3] Biosensors and Bioelectronics Volume 144, 1 November 2019, 111697.

[4] Electrochimica Acta Volume 319, 1 October 2019, Pages 375-381.

[5] Coordination Chemistry Reviews Volume 400, 1 December 2019, 213041.

[6] Acs Nano 2019, 13 (1), 284-294.

[7] Adv. Sci. 2018, 5, 1800518.

[8] ACS Appl. Mater. Interfaces 2017, 9, 40077-40086.

[9] J. Am. Chem. Soc. 2017, 139, 16235-16247.

[10] Nanophotonics 2020; 9(8): 2233–2249.

[11] Nanophotonics 2020; 9(8): 2233–2249.

[12] Nanoscale 2017;9:17859–6.

[13] Materials Science and Engineering: C Volume 98, May 2019, Pages 874-886.

[14] Materials Science and Engineering: C Volume 116, November 2020, 111212.

[15] ACS Nano 2017, 11, 12, 12696–12712.

[16] Angew. Chem. Int. Ed. 2020, 59, 6601 –6606.

[17] ACS Nano 2019, 13, 2, 1499–1510.

[18] Materials Science and Engineering: C Volume 98, May 2019, Pages 874-886.

**Supplementary Table S7.** Some applications of MXene-based immunoengineered nanosystems for combination therapy.

| **Immunoengineered MXene Nanosystems** | **Photodynamic Therapy**  **(PDT)-Assisted Combined Therapy** | ***In vitro/vivo* Models** | **Reference** |
| --- | --- | --- | --- |
| Ti_3_C_2_ MXene Composite (MXene-Doxorubicin) | at 808 nm laser, 0.8 W/cm^2^, 10 min,  ROS type: O_2_  NIR-induced PDT Efficiency of 58.3% | *In vitro* at 42 μg/mL  2.0 mg/kg | [1] |
| Ti_3_C_2_ MXene Composite (MXene-Iron oxide NPs-Glucose oxidase) | at 808 nm laser  ROS type: singlet oxygen generation (^1^O_2_)  Improved PDT efficiency of 27.27% | *In vitro*  / *In vivo* | [2] |

[1] ACS Appl. Mater. Interfaces 2017, 9, 40077–40086

[2] Theranostics, 2018, 8, 92–108

**Supplementary Table S8.** Application of MXene based nanosystems for photo-thermal therapy applications.

| **MXene Types and Nanosystems** | **Photo-thermal Conversion Efficiency (ƞ)** | **Reported Anti-Cancer Applications** | **Study Model** | **Ref.** |
| --- | --- | --- | --- | --- |
| Ti_3_C_2_ nanosheets | 30.6% | Photothermal ablation of tumor | *In vitro*-4T1 murine breast cancer cell line  *In vivo*-Kunming mice model | [1] |
| Ti_3_C_2_-DOX | 58.3% | Synergistic PTT/PDT/chemotherapy | *In vitro*  - HCT-116 (human colorectal carcinoma cell line)  -A2780 (human ovarian cancer cell line)  *In vivo*-Athymic nude mice model | [2] |
| Ti_3_C_2-_Au nanocomposite | 34.3% in NIR-I, 39.6% in NIR-II biowindows | Photoacoustic and computed tomography dual-modal imaging, and radiotherapy by improving the tumor oxygenation | *In vitro-*4T1 murine breast cancer cell line  *In vivo*-4T1 tumor bearing mice | [3] |
| Ti_3_C_2_-CoNW (Cobalt Nanowire) heterojunction | 34.42% | Dual stimuli-responsive drug release, and chemo-photothermal therapy. | *In vitro*-4T1 murine breast cancer cell line | [4] |
| Ti_3_C_2-_POM _(_Polyoxometalate) | 21.9% | Guidance for Diagnostic-imaging and tumor hyperthermia nanotherapy monitoring | *In vitro* 4T1 murine breast cancer cell line  *In vivo*-4T1 tumor-bearing mice | [5] |
| MnOx/ Ti_3_C_2_−SP composite nanosheets | 22.9% | Tumor ablation and tumor-growth suppression | *In vitro-*4T1 murine breast cancer cell line  *In vivo*-4T1 breast tumor-bearing mice | [6] |
| Ti_3_C_2_-Quantum Dots | 52.2% | PTT | *In vitro-* HeLa, MCF-7, U251 and HEK 293 cells  *In vivo-*Balb/c nude mice | [7] |
| Ti_2_C-PEG nanosheets | 87.1% | PTT | *In vitro-*  A375 (human skin malignant melanoma cells), HaCaT (human immortalized keratinocytes), MCF-7 (human breast cancer cells) and MCF-10A (normal human mammary epithelial cells) | [8] |
| Ti_2_N Quantum Dots | 48.62% and 45.51% in NIR-I and NIR-II biowindows | Photoacoustic (PA) imaging-guide PTT | *In vitro-*4 T1, U87, and 293 T cells  *In vivo-* Female Balb/c mice | [9] |
| Ta_4_C_3_-SP (Soybean phospholipid)  nanosheets | 44.7% | Photothermal ablation of tumor | *In vitro-* 4T1 murine breast cancer cell line  *In vivo-* 4T1 breast tumor-bearing mice | [10] |
| MnOx/ Ta_4_C_3_–SP | 34.9% | Photoacoustic (PA) imaging and photothermal therapy (PTT) for tumor-growth suppression | *In vitro*-4T1 murine breast cancer cell line  *In vivo*-Kunming mice model | [11] |
| Ta_4_C_3_-IONP-SPs  (iron-oxide functionalization composite MXene) | 32.5% | Breast-cancer theranostic | *In vitro*-4T1 murine breast cancer cell line  *In vivo*- Female Kunming mice and BALB/c nude mice | [12] |
| Nb_2_C-PVP nanosheets | 36.4% at NIR-I and 45.65% at NIR-II biowindows | Photothermal ablation and eradication of tumor in both NIR-I and NIR-II biowindows | *In vitro*-4T1 murine breast cancer cell line  *In vivo*-Female Kunming mice | [13] |
| CTAC-Nb_2_C-MSN-PEG-RGD | 28.6% | PTT and Chemotherapy | *In vitro-* U87 murine neuroglioma cells-  *In vivo-* Tumor-bearing nude mice | [14] |

[1] Nano letters 17, no. 1 (2017): 384-391.

[2] ACS applied materials & interfaces 9, no. 46 (2017): 40077-40086.

[3] ACS nano 13, no. 1 (2018): 284-294.

[4] Materials Science and Engineering: C 116 (2020): 111212.

[5] Nano Research 11, no. 8 (2018): 4149-4168.

[6] Chemistry of Materials 29, no. 20 (2017): 8637-8652.

[7] Nanoscale 9, no. 45 (2017): 17859-17864.

[8] Materials Science and Engineering: C 98 (2019): 874-886.

[9] Chem Eng J. 2020;400:126009.

[10] Advanced Materials 30, no. 4 (2018): 1703284.

[11] ACS nano 11, no. 12 (2017): 12696-12712.

[12] Theranostics 8, no. 6 (2018): 1648.

[13] Journal of the American Chemical Society 139, no. 45 (2017): 16235-16247.

[14] Theranostics 8, no. 16 (2018): 4491.

**Supplementary Table S9.** List of human quantitative PCR primers used in this study.

| **Human Gene** | **Strand** | **Sequence (5’ to 3’)** |
| --- | --- | --- |
| IRF1 | *s* | CCT CCA CCT CTG AAG CTA CAA C |
|  | *as* | CCA TCC ACG TTT GTT GGC TG |
| TAP1 | *s* | TCG TTG TCA GTT ATG CAG CG |
|  | *as* | AAT GGC CAT CTC CCC AAG AG |
| CDH5 | *s* | CTT CAC CCA GAC CAA GTA CAC A |
|  | *as* | AAT GGT GAA AGC GTC CTG GT |
| VCAM1 | *s* | GGA AAT GAC CTT CAT CCC TAC CA |
|  | *as* | ATC TCT GGG GGC AAC ATT GA |
| PECAM1 | *s* | GCT GAC CCT TCT GCT CTG TT |
|  | *as* | ATC TGG TGC TGA GGC TTG AC |
| SELE | *s* | CCG AGC GAG GCT ACA TGA AT |
|  | *as* | GCA TCG CAT CTC ACA GCT TC |
| CCL2 | *s* | AGA TCT GTG CTG ACC CCA AG |
|  | *as* | GGA GTT TGG GTT TGC TTG TCC |
| CXCL9 | *s* | GGT GTT CTT TTC CTC TTG GGC |
|  | *as* | TTC TCA CTA CTG GGG TTC CTT G |
| CXCL10 | *s* | AAG TGG CAT TCA AGG AGT ACC T |
|  | *as* | GGA CAA AAT TGG CTT GCA GGA |
| HLA-A | *s* | GAG TAT TGG GAC CAG GAG ACA C |
|  | *as* | CCA CGT CGC AGC CAT ACA TTA |
| B2M | *s* | GAT GAG TAT GCC TGC CGT GT |
|  | *as* | CTG CTT ACA TGT CTC GAT CCC A |
| FASLG | *s* | CTA CCA GCC AGA TGC ACA CA |
|  | *as* | CCT TGA GTT GGA CTT GCC TGT |
| PD-L1 | *s* | CCT CTG GCA CAT CCT CCA AAT |
|  | *as* | GCT GGA TTA CGT CTC CTC CAA |
| PD-L2 | *s* | TCC AAC TTG GCT GCT TCA CA |
|  | *as* | CCA CAG GTT CAG ATA GCA CTG T |
| CD86 | *s* | CGA CGT TTC CAT CAG CTT GTC |
|  | *as* | TCC AAG GAA TGT GGT CTG GG |
| GAPDH | *s* | ACA GTT GCC ATG TAG ACC |
|  | *as* | TTG AGC ACA GGG TAC TTT A |
| ACTB | *s* | CTT CGC GGG CGA CGA T |
|  | *as* | CCA CAT AGG AAT CCT TCT GAC C |

**Supplementary Table S10.** List of western blotting antibodies used in this study.

| **Antibody Name** | **Vendor** | **Catalog Number** | **Dilution** |
| --- | --- | --- | --- |
| Anti-HLA-DRα antibody | Santa Cruz Biotechnology | sc-55592 | 1:200 |
| Anti-ICAM1 antibody | abcam | ab53013 | 1:2,000 |
| HRP anti-β-actin antibody | Santa Cruz Biotechnology | sc-47778 | 1:10,000 |
| HRP anti-mouse secondary | Bio-Rad Laboratories | 1706516 | 1:10,000 |
| HRP anti-rabbit secondary | Bio-Rad Laboratories | 1706515 | 1:4,000 |

**Supplementary Table S11.** List of flow cytometry antibodies used in this study.

| **Antibody Name** | **Clone** | **Vendor** | **Catalog Number** | **Quantity**  **(for 10^6^ cells in 100 µL)** |
| --- | --- | --- | --- | --- |
| FITC anti-human CD4 | RPA-T4 | BioLegend | 300506 | 2 µg |
| PE anti-human CD3 | UCHT1 | BioLegend | 300456 | 0.5 µg |
| PerCP anti-human IFN-γ | 4S.B3 | BioLegend | 502524 | 0.5 µg |
| AF647 anti-human IL-4 | 8D4-8 | BioLegend | 500712 | 0.25 µg |
| FITC mouse IgG1κ isotype | MOPC-21 | BioLegend | 400107 | 2 µg |
| PE mouse IgG1κ isotype | MOPC-21 | BioLegend | 400111 | 0.5 µg |
| PerCP mouse IgG1κ isotype | MOPC-21 | BioLegend | 400147 | 0.5 µg |
| AF647 mouse IgG1κ isotype | MOPC-21 | BioLegend | 400135 | 0.25 µg |

**Supplementary Table S12.** List of immunocytochemistry antibodies used in this study**.**

| **Antibody Name** | **Vendor** | **Catalog Number** | **Dilution** |
| --- | --- | --- | --- |
| Mouse anti-rat CD4a | Cedarlane | CL003AP | 1:100 |
| Mouse anti-rat CD8a | Cedarlane | CL004AP | 1:100 |
| Goat anti-human/mouse/rabbit/rat α-SMA | Novus Biologicals | NB300-978 | 1:300 |
| Mouse anti-human vWF | abcam | ab201336 | 1:200 |
| Rabbit anti-human VE cadherin | abcam | ab33168 | 1:100 |
| AF488 goat anti-mouse secondary | Invitrogen | A-11017 | 1:500 |
| AF647 goat anti-rabbit secondary | Invitrogen | A-21246 | 1:500 |
| AF647 goat anti-mouse secondary | Invitrogen | A-21237 | 1:500 |
| AF647 donkey anti-goat secondary | abcam | ab150135 | 1:500 |

**Supplementary Table S13.** List of rat quantitative PCR primers used in this study.

| **Rat Gene** | **Strand** | **Sequence (5’ to 3’)** |
| --- | --- | --- |
| CD3E | *s* | TGT GAC CCG AGG AAC TGG TA |
|  | *as* | AGA ATA CAG GTC CCG TTG GC |
| CD4 | *s* | AAA GTG ACT CAG CCC GAC AG |
|  | *as* | GTA GAC ATT GCC ACA CCC CT |
| CD8A | *s* | ACT AGC TGG TGC AGG GAT CT |
|  | *as* | GCA CTG CTA AGG GCA GTT CT |
| CD28 | *s* | CCC GCT GCT TGT GGT AGA TA |
|  | *as* | TGT AAA GGG ATG CCC GGA AC |
| TFRC | *s* | AGC TGC CAC CTG AGA ACA TC |
|  | *as* | CGC ACG CCC TTT ATT CAT GG |
| GAPDH | *s* | CCC CAA CAC TGA GCA TCT CC |
|  | *as* | GTA TTC GAG AGA AGG GAG GGC |
